# Supplementary material for: Circulating microRNAs in Cerebrospinal Fluid and Plasma: Sensitive Tool for Detection of Secondary CNS Involvement, Monitoring of Therapy and Prediction of CNS Relapse in Aggressive B-NHL Lymphomas
Source: Cancers (Basel). 2022 May 6;14(9):2305. doi: 10.3390/cancers14092305 (PMC9103209; doi:10.3390/cancers14092305)
Supplement: Supplementary file 1 [file cancers-14-02305-s001.zip › cancers-1659423-supplementary.pdf]

Supplementary information for:

Circulating microRNAs in cerebrospinal fluid and plasma: sensitive tool for detection of CNS involvement, monitoring of therapy and prediction of CNS relapse in aggressive B-NHL

Pavle Krsmanovic, Heidi Mocikova, Kamila Chramostova, Magdalena Klanova, Marie Trnkova, Michal Pesta, Peter Laslo, Robert Pytlik, Tomas Stopka, Marek Trneny and Vit Pospisil [1]

[1]To whom correspondence should be addressed. E-mail: vitek_pos@hotmail.com

Content:

Supplementary text p. 2
Supplementary methods p. 2
Supplementary tables p. 4
Supplementary figures p. 10

Supplementary text:

Multivariate regression analysis (MVA) and correlation with clinical characteristics:
We employed a multivariate analysis to evaluate the effect of oncomiRs, clinical and biochemical characteristics and prognostic indices on lymphoma CNS involvement (Suppl. methods and Suppl. table 5). While oncomiR-indices (as well as individual oncomiRs) were significantly associated with CNS lymphoma involvement (when compared to systemic lymphoma, see Table 1 *P* values), there was no significant effect of either the recorded clinical characteristics or the prognostic indices on CNS lymphoma involvement. In addition, none of them had an effect on the oncomiR-indices, indicating that oncomiRs are an independent prediction factor. However, we observed an enrichment of certain clinical characteristics and prognostic indices in SCNSL compared to the cohorts of systemic patient (see Suppl. table 4 for details).
We observed a partial correlation of oncomiR-indices with FCM positive examinations of CSF in all lymphoma subtypes in CSF (r= 0.37 - 0.82, Pearson correlation). In MCL a certain level of correlation of oncomiR-indices with MIPI prognostic index (CSF r = 0.58, plasma r = 0.4) and CSF LDH levels (r = 0.82) was observed.
In addition, a positive but low to moderate correlation of oncomiR-indices levels with circulating lymphoma phase (presence of circulating lymphoma cells at peripheral blood), bone marrow involvement and proliferative index was observed as follows: Circulating lymphoma phase (CSF / plasma): DLBCL (r=0.16 / 0.05), MCL (r=0.23 / 0.05), BL (r= 0.48 / 0.46). Bone marrow involvement (CSF / plasma): DLBCL (r=0.25 / 0.1), MCL (r=0.2 / 0.16), BL (r= 0.58 / 0.3). Proliferative index: DLBCL (r=0.19 / 0.1), MCL (r=0.35 / 0.35), BL (r= 0.2 / 0.25). Categorization of oncomiR-indices (above / below threshold (Tab.1)) increased the correlation coefficient by ~10-20% in CSF and 50-100% in plasma.

Summary of the possible use of cerebrospinal fluid and plasma to detect CNS lymphoma:
i. CSF oncomiRs are suitable for early detection of CNS lymphoma involvement in all studied B-NHL subtypes. Plasma oncomiRs are good markers for detection of CNS involvement in BL (in MCL and DLBCL with lower accuracy). **ii.** Both CSF and plasma oncomiRs are suitable for monitoring of therapy efficacy (all dg). **iii.** Increasing oncomiR levels in both CSF and plasma indicate upcoming CNS relapse. **iv.** OncomiRs in plasma (but not CSF) are markers for prediction of CNS relapse risk at diagnosis of systemic DLBCL (combined CNS-IPI/oncomiR prediction model) and in both CSF and plasma are predictive for overall survival in DLBCL.

Supplementary methods:

MicroRNAs (Assay ID): hsa-miR-21-5p (000397), hsa-miR-19a-3p (000395), hsa-miR-20a-5p (000580), hsa-miR-92a-3p (000431), hsa-miR-155-5p (002623), hsa-let-7a-5p (000377), cel-miR-39-3p (000200) (all Applied Biosystems, Thermo Fisher).
Panel for initial screening included following candidate microRNAs, selected by a compilation of published data of microRNA expression in CNS involving B-NHL: miR-9-5p (000583), miR-15a-5p (000389), miR-16-5p (000391), miR-17-5p (000393), miR-18a-3p (002423), miR-19a-3p (000395), miR-20a-5p (000580), miR-21-5p (000397), miR-24-3p (000402), miR-25-3p (000403), miR-30c-5p (000419), miR-30d-5p (000420), miR-92a-3p (000431), miR-101-3p (002253), miR-106a-5p (002169), miR-106b-5p (000442), miR-142-5p (000465), miR-155-5p (002623), miR-210-3p (000512), miR-223-3p (002295), miR-let7a-5p (000377), RNU44 (001094). miR-let7a-5p, miR-16-5p, miR-24-3p, RNU44 were tested for normalization, best performing let-7a-5p was used [18, 40]. Sequences are available at Applied Biosystems, Thermo Fisher www under the respective assay ID.

OncomiR-index/-ices calculation:
In order to determine the coefficients for oncomiRs-indices, we have first calculated the probability of CNS involvement for each sample according to the following formula:
*Probability [CNS involvement] = 1 / [1 + exp{X - sum (Coefficient[miRi] * Abundance[miRi]) }]*
Initially, each coefficient was set to 1 and the X value to 0.5. The oncomiRs abundances used for the probability calculation were miR-let-7a-normalized, and equalized to average of Ctrl and average of respective systemic lymphoma samples. These scaled values were used in order to assess the relative impact of each oncomiR and not favor primarily those oncomiRs with higher absolute abundance levels and/or disfavor those with lower absolute levels.
Subsequently, the average values of probabilities of CNS positive and systemic samples were calculated. Next, the coefficients which maximize the difference between average probabilities of CNS involvement in CNS positive and systemic lymphoma were then determined using the solver statistical tool (MS Excel). The upper limits of coefficients were initially set to 2 (for X to 10) and if any coefficients reached the upper limit, the solver tool would be rerun only on those coefficients without the maximal value limitation. Finally, determined coefficients were used for calculation of oncomiR-indices by regression the formula:
OncomiR-index = sum (Coefficient[miRi] * Abundance[miRi]).
For each lymphoma subtype, the procedure was repeated for multiple oncomiRs combinations. The combination which yielded the best separation of CNS involving and systemic samples, i.e. highest ROC area, specificity and sensitivity, was finally used.

The stratification of patients for Kaplan-Meier and Cox models to estimate relapse risk and overall survival:

1. oncomiRs thresholds for stratification of microRNA (oncomiR) high/low (as in Table 1, determined by ROC as described in method section):

**Test:**	**oncomiR-index**	**threshold (log_2)**	
CNS relapse subsequent (plasma)	miR-21	3.18	(1.67)
CNS relapse current (CSF)	miR-21/20a/155	8.42	(3.07)
CNS relapse current (plasma)	miR-21/19a/20a/155	6.13	(2.62)
Overall survival (CSF)	miR-21/20a/155	8.42	(3.07)
Overall survival (plasma)	miR-21/19a/20a/155	6.13	(2.62)

2. CNS-IPI model stratification (by CNS-IPI score)**:**

High:	(4-6)
Intermediate:	(2-3)
Low:	(0-1)

3. CNS-IPI/oncomiR model stratification:

Two risks:	(high microRNA and high CNS-IPI)
One risk:	(either high microRNA or high CNS-IPI)
No risk:	(neither microRNA nor CNS-IPI are high)

For estimation of relapse risk and OS only patients having oncomiR values in both CSF and plasma were included.

Multivariate regression analysis:

In multivariate analysis, the following clinical parameters were tested (in addition to oncomiRs): clinical stage, ECOG, Performance status, IPI, aaIPI, CNS-IPI, MIPI, number of extra nodal lymphoma sites, involvement of the kidneys and adrenal glands, bone marrow and biochemical analysis of CSF and plasma as indicated in Suppl. table 5. Data on cell-of-origin (GCB/non-GCB) and MYC and BCL2 expression and rearrangements were not available in a sufficient number of patients to be employed in MVA.

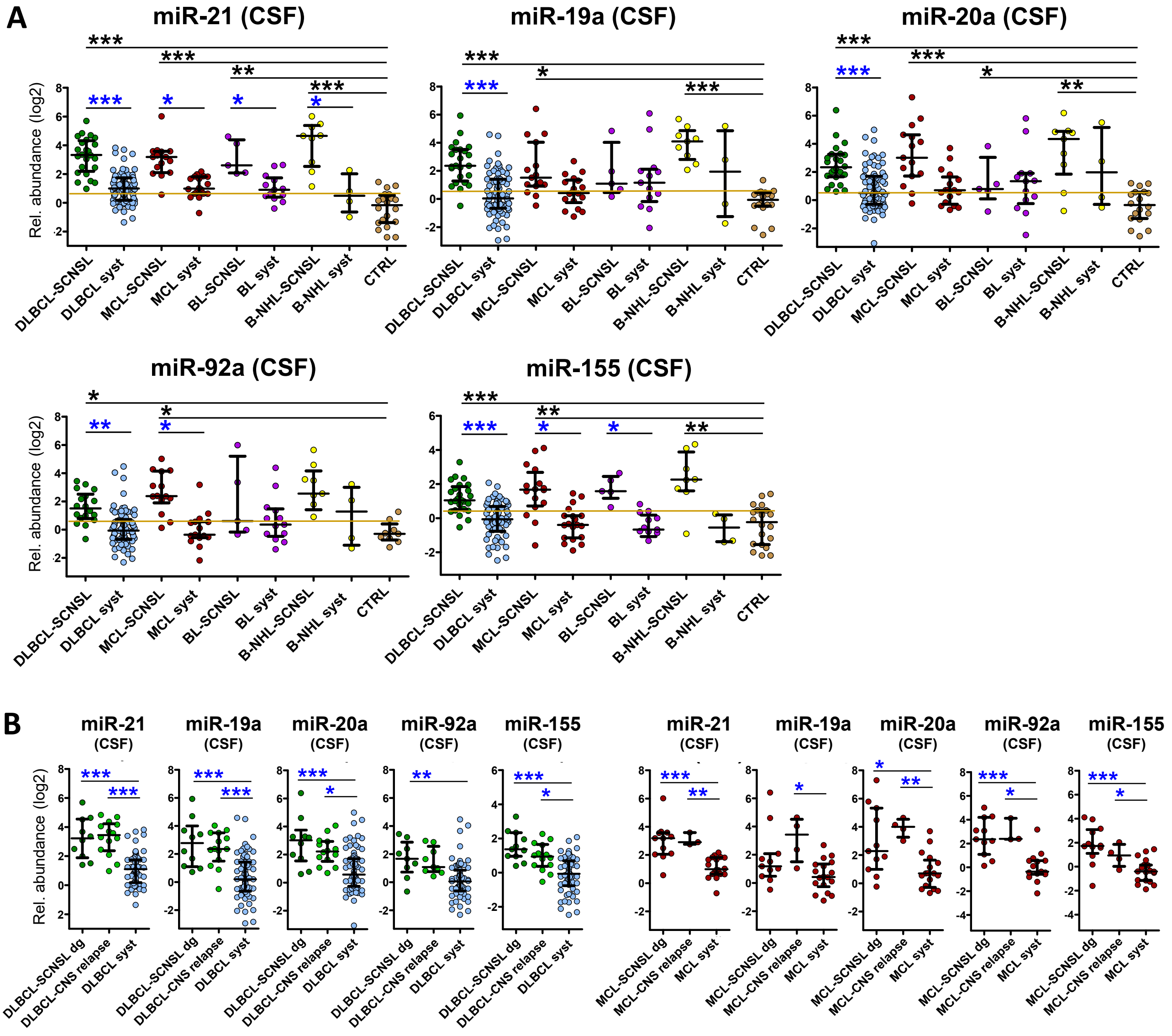

Figure S1. Abundance of oncogenic microRNAs in cerebrospinal fluid of CNS involving lymphomas.
Cerebrospinal fluid (CSF) levels of miR-21/-19a/-20a/-92a/-155 of (**A**) lymphoma patients of indicated B-NHL diagnoses with secondary CNS involvement (SCNSL), compared to systemic lymphoma (syst) and controls with neurological disorders (CTRL). B-NHL denotes B-NHL-NOS (not otherwise specified, high grade).
(**B**) DLBCL-SCNSL (left panel) and MCL-SCNSL (right panel) are subdivided to lymphoma with secondary CNS involvement presented at the time of diagnosis (SCNSL dg) and newly detected CNS relapses. qRT-PCR. Values are equalized to the average of CTRL. Log2 scale. The yellow line marks the 99.9% confidence interval of CTRL. Median ± interquartile range, *P<0.05, **P<0.01, *** P<0.001, Kruskal-Walliss test.

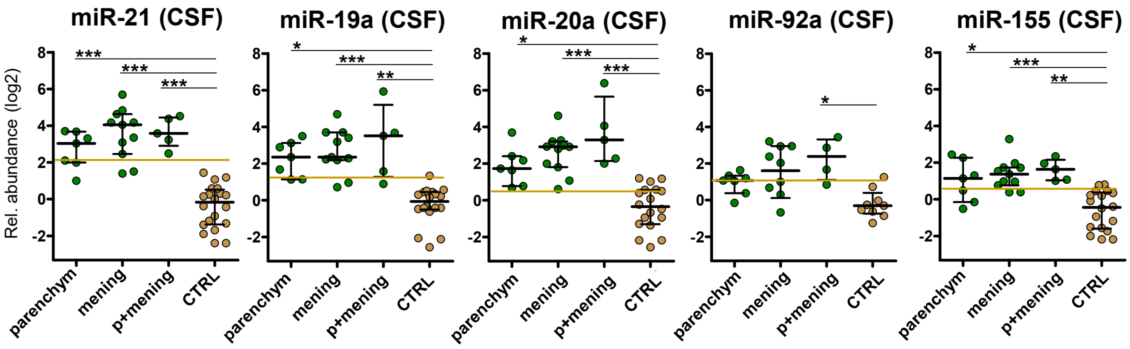

Figure S2. Abundance of oncomiRs in CSF of DLBCL stratified by parenchymal and meningeal involvement.
Levels of indicated microRNAs in the cerebrospinal fluid (CSF) of secondary CNS DLBCL (DLBCL-SCNSL) divided according to parenchymal (parenchym), leptomeningeal (mening) and combined (p+mening) CNS involvement. The yellow line marks the 99.9% confidence interval for CTRL. Values are equalized to average value of CTRL. Log2 scale. Median ± interquartile range, *P<0.05, **P<0.01, *** P<0.001, Kruskal-Walliss test.

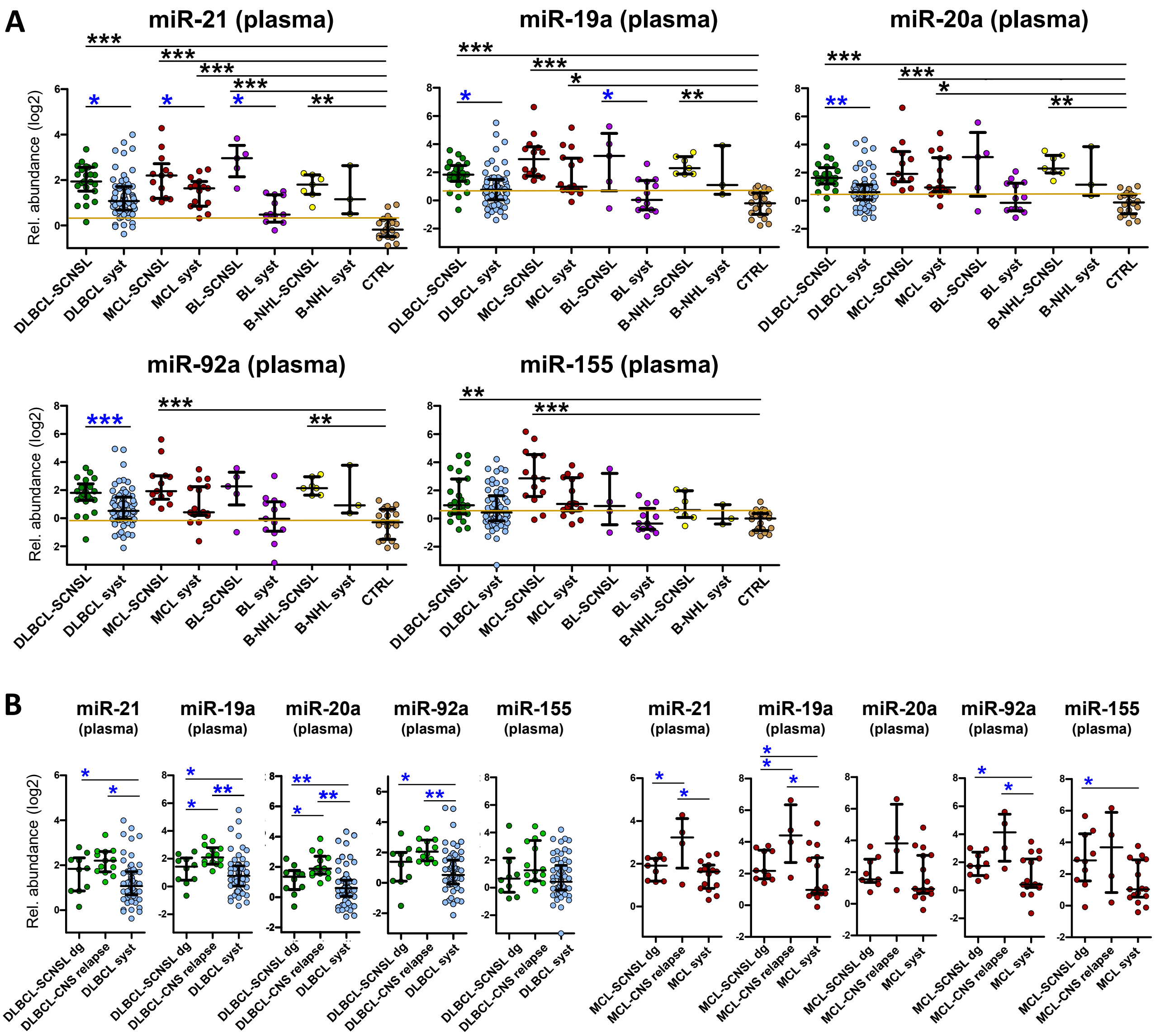

Figure S3. Abundance of oncomiRs is increased in plasma of B-NHL involving CNS.
Plasma levels of miR-21/-19a/-20a/-92a/-155 of (**A**) lymphoma patients with indicated B-NHL diagnoses, with and without CNS involvement, and controls (CTRL). B-NHL denotes B-NHL-NOS (not otherwise specified, high grade).
(**B**) DLBCL-SCNSL (left panel) and MCL-SCNSL (right panel) are subdivided to lymphoma with secondary CNS involvement presented at the time of diagnosis (SCNSL dg) and CNS relapses. Values are equalized to average of CTRL. Log2 scale. The yellow line marks 99.9% confidence interval of CTRL. Median ± interquartile range, *P<0.05, **P<0.01, *** P<0.001, Kruskal-Walliss test.

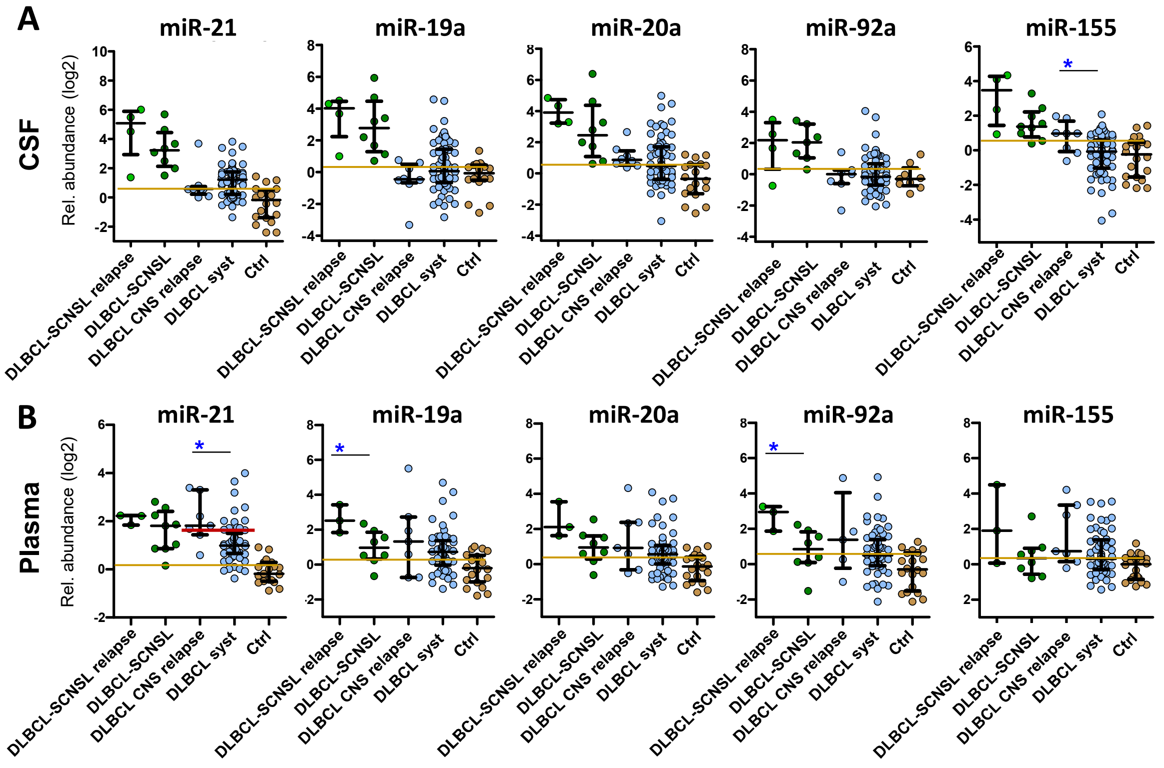

Figure S4. Abundance of oncomiRs in DLBCL patients with subsequent CNS relapse.
Levels of miR-21/-19a/-20a/-92a/-155 in DLBCL patients at the time of initial diagnosis, who subsequently developed CNS relapse. Abbreviation: DLBCL-SCNSL relapse: DLBCL with secondary CNS involvement which developed CNS relapse; DLBCL-SCNSL: non-relapsing group; DLBCL CNS relaps: systemic lymphoma with subsequent relapse/progression to CNS; DLBCL syst: systemic non-relapsing group; CTRL: control patients. CSF (**A**) and plasma (**B**). The yellow line marks the 99.9% confidence interval for controls. The red line (plasma miR-21) indicates the threshold for prediction of CNS relapse. Y-axis values are equalized to avarage of controls. Log2 scale. Median ± interquartile range, *P<0.05, **P<0.01, *** P<0.001, Mann-Whitney test.

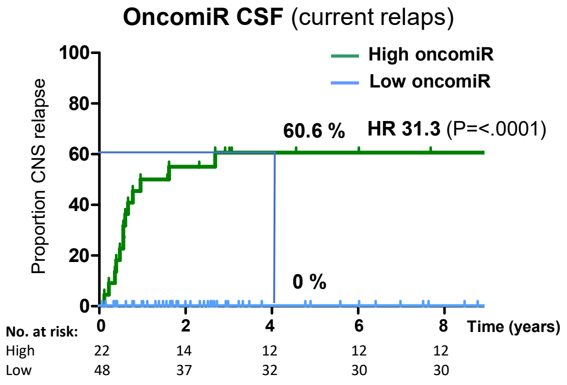

oncomiR CSF	CNS relapse	No relapse	All patients	Relapse rate	HR	95% CI	*P*
High	14 (100.0%)	7 (12.5%)	21 (30.0%)	60.6%	31.3	9.8 - 99.8	<0.0001
Low	0 (0.0%)	49 (87.5%)	49 (70.0%)	0.0%			

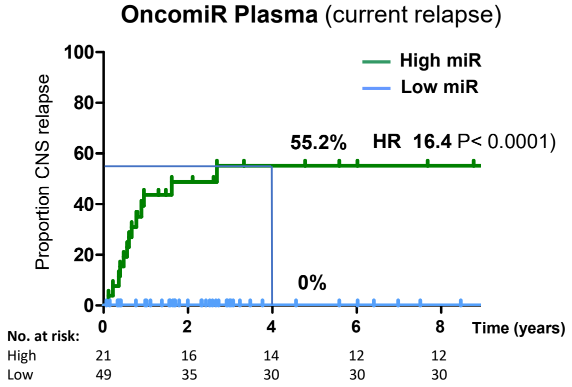

oncomiR plasma	CNS relapse	No relapse	All patients	Relapse rate	HR	95% CI	*P*
High	14 (100.0%)	12 (21.4%)	26 (37.1%)	55.2%	16.5	5.5 - 49.4	<0.0001
Low	0 (0.0%)	44 (78.6%)	44 (62.9%)	0.0%			

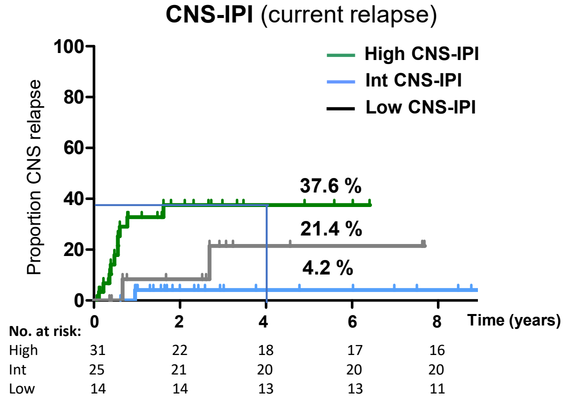

CNS-IPI	CNS relapse	No relapse	All patients	Relapse rate	HR	95% CI	*P*
High	10 (71.4%)	21 (37.5%)	31 (44.3%)	37.6%	High vs Int./ Low		
Int	2 (14.3%)	23 (41.1%)	25 (35.7%)	4.2%	5.8	1.9 - 18.9	0.0038
Low	2 (14.3%)	12 (21.4%)	14 (20.0%)	21.4%	2.4	0.73 - 7.7	0.1536
				HR Int. vs Low	0.2	0.2 - 2.5	0.2286

Figure S5. Prognostic value of circulating oncomiRs in DLBCL at the time of CNS relapse.
Risk for CNS relapse stratified by oncomiR levels in cerebrospinal fluid (CSF) or plasma at the time of CNS relapse (current relapse) and by CNS-IPI score (Kaplan-Meier estimates). DLBCL systemic patients were stratified by high / low microRNA levels acquired at the time of CNS relapse (current relapse, upper panels) or by CNS-IPI (lower panel). Note: HR, 95% CI and P in the tables below charts were obtained from univariate models of indicated categories. Abbreviations: CNS, central nervous system; IPI, International Prognostic Index; Int., intermediate; HR, hazard ratio. For details on risk stratification see Suppl. methods.

Figures S6, S7, S8 follow behind Supplementary tables starting at page 15.

p 15-18 :

Figure S6. Receiver operating analysis of oncomiRs in CSF of CNS lymphoma compared to controls or systemic lymphoma.
Receiver operating characteristic (ROC) curves for the possibility of indicated oncomiRs in CSF to discriminate between CNS lymphoma and controls (left panels) and between CNS involving and systemic lymphoma (right panels) in: (**A**) DLBCL, (**B**) MCL, (**C**) BL and (**D**) B-NHL-NOS. The X-axes show the percent of specificity and the Y-axes denote percent of sensitivity (100-sesintivity).

p 19-22:

Figure S7. Receiver operating analysis of oncomiRs in plasma of CNS lymphoma compared to controls or systemic lymphoma.
Receiver operating characteristic (ROC) curves for the possibility of indicated oncomiRs in plasma to discriminate between CNS lymphoma and controls (left panels) and between CNS involving and systemic lymphoma (right panels) in: (**A**) DLBCL, (**B**) MCL, (**C**) BL and (**D**) B-NHL-NOS. The X-axes show the percent of specificity and the Y-axes denote percent of sensitivity (100-sesintivity).

P 23:
Figure S8. Receiver operating analysis of oncomiR-indices in CSF (A) and plasma (B) of CNS lymphoma compared to control samples.

A

CSF	CNS vs.	miR-21		miR-19a		miR-20a		miR-92a		miR-155		oncomiR-index		
		AUC	p-value	AUC	p-value	AUC	p-value	AUC	p-value	AUC	p-value	miR combination	AUC	p-value
DLBCL-SCNSL	CTRL	**0.99**	< 0.0001	**0.96**	< 0.0001	**0.97**	< 0.0001	**0.91**	0.0011	**0.91**	< 0.0001	**miR-21/-20a/-155**	**1.00**	< 0.0001
	Syst	**0.91**	< 0.0001	**0.86**	< 0.0001	**0.81**	< 0.0001	**0.85**	< 0.0001	**0.85**	< 0.0001	**miR-21/-20a/-155**	**0.96**	< 0.0001
MCL-SCNSL	CTRL	**0.98**	< 0.0001	**0.89**	0.0001	**0.93**	< 0.0001	**0.97**	0.0003	**0.89**	0.0001	**miR-21/-20a/-92a/-155**	**0.99**	0.0003
	Syst	**0.95**	< 0.0001	**0.82**	0.0009	**0.84**	0.0013	**0.90**	0.0002	**0.92**	< 0.0001	**miR-21/-20a/-92a/-155**	**0.93**	< 0.0001
BL-SCNSL	CTRL	**1.00**	0.0007	**0.88**	0.0102	**0.79**	0.0527	**0.84**	0.0482	**0.98**	0.0015	**miR-21/-155**	**1.00**	0.0007
	Syst	**0.92**	0.0067	**0.54**	0.8054	**0.52**	0.8825	**0.62**	0.4598	**0.98**	0.0022	**miR-21/-155**	**1.00**	0.0014
B-NHL-NOS-SCNSL	CTRL	**0.99**	< 0.0001	**1.00**	< 0.0001	**0.90**	0.0008	**0.88**	0.0071	**0.93**	0.0007	**miR-21/-155**	**0.94**	0.0002
	Syst	**0.94**	0.0175	**0.69**	0.3082	**0.63**	0.4969	**0.69**	0.3082	**0.93**	0.0234	**miR-21/-155**	**0.94**	0.0136

B

Plasma	CNS vs.	miR-21		miR-19a		miR-20a		miR-92a		miR-155		oncomiR-index		
		AUC	p-value	AUC	p-value	AUC	p-value	AUC	p-value	AUC	p-value	miR combination	AUC	p-value
DLBCL-SCNSL	CTRL	**0.98**	< 0.0001	**0.94**	< 0.0001	**0.94**	< 0.0001	**0.91**	< 0.0001	**0.83**	0.0002	**miR-21/-19a/-20a/-155**	**0.94**	< 0.0001
	Syst	**0.79**	< 0.0001	**0.79**	< 0.0001	**0.81**	< 0.0001	**0.76**	0.0004	**0.66**	0.0187	**miR-21/-19a/-20a/-155**	**0.79**	< 0.0001
MCL-SCNSL	CTRL	**1.00**	< 0.0001	**1.00**	< 0.0001	**0.98**	< 0.0001	**0.98**	< 0.0001	**0.93**	< 0.0001	**miR-21/-19a/-155**	**1.00**	< 0.0001
	Syst	**0.72**	0.0503	**0.75**	0.0200	**0.67**	0.1145	**0.77**	0.0150	**0.74**	0.0276	**miR-21/-19a/-155**	**0.84**	0.0016
BL-SCNSL	CTRL	**1.00**	0.0007	**0.88**	0.0109	**0.83**	0.0250	**0.86**	0.0145	**0.74**	0.1412	**miR-21/-19a/-155**	**1.00**	0.0020
	Syst	**1.00**	0.0016	**0.82**	0.0385	**0.75**	0.1039	**0.80**	0.0546	**0.71**	0.2130	**miR-21/-19a/-155**	**1.00**	0.0033
B-NHL-NOS-SCNSL	CTRL	**0.98**	0.0005	**1.00**	0.0003	**1.00**	0.0001	**1.00**	0.0001	**0.77**	0.0356	**miR-19a/-155**	**1.00**	0.0001
	Syst	**0.61**	0.6056	**0.67**	0.4386	**0.67**	0.4251	**0.67**	0.4251	**0.71**	0.3051	**miR-19a/-155**	**0.67**	0.4251

Table S1. Summary of Receiver operating characteristics (ROC) of oncomiRs in CSF and plasma. Comparison of area under curve (AUC) of indicated CNS involving lymphoma vs. control (grey rows) or CNS vs. systemic lymphoma (white rows). **Left panel**: the single oncomiRs (from Suppl. fig. 5 and 6), **right panel**: oncomiR-indices (from Fig. 4). (**A**) CSF, (**B**) plasma. The area under curve (AUC) is a measure of how well the respective oncomiR distinguishes between CNS lymphoma from CTRL or systemic lymphoma. Higher value = better separation, ROC=1 denotes absolute separation. OncomiR-index combines expression of individual oncomiRs (best combination) to one variable, yielding higher specificity/sensitivity and separation than individual oncomiR.

A CSF

CSF

CNS vs Ctrl - single oncomiR / oncomiR-index

Sensitivity

CNS lymphoma	miR-21	miR-19a	miR-20a	miR-92a	miR-155	Index	Percent
DLBCL	95.8	100.0	100.0	94.4	88.0	-21/20a/155	100.0
MCL	93.3	80.0	80.0	100.0	81.3	-21/20a/92a/155	93.3
BL	100.0	80.0	80.0	80.0	100.0	-21/155	100.0
B-NHL-NOS	100.0	100.0	77.8	88.9	87.5	-21/155	88.9

Specificity

	miR-21	miR-19a	miR-20a	miR-92a	miR-155	Index	Percent
DLBCL	95.0	94.4	83.3	87.5	77.8	-21/20a/155	100.0
MCL	100.0	94.4	100.0	87.5	88.2	-21/20a/92a/155	100.0
BL	100.0	94.4	81.3	75.0	88.2	-21/155	100.0
B-NHL-NOS	90.0	100.0	100.0	88.9	100.0	-21/155	100.0

Youden index

	miR-21	miR-19a	miR-20a	miR-92a	miR-155	Index	Percent
DLBCL	90.8	94.4	83.3	81.9	65.8	-21/20a/155	100.0
MCL	93.3	74.4	80.0	87.5	69.5	-21/20a/92a/155	93.3
BL	100.0	74.4	61.3	55.0	88.2	-21/155	100.0
B-NHL-NOS	90.0	100.0	77.8	77.8	87.5	-21/155	88.9

B Plasma

Plasma

Sensitivity

	miR-21	miR-19a	miR-20a	miR-92a	miR-155	Index	Percent
DLBCL	95.7	83.3	91.7	81.8	79.2	-21/19a/20a/155	91.3
MCL	100.0	100.0	92.3	92.3	85.7	-21/19a/155	100.0
BL	100.0	80.0	80.0	80.0	75.0	-21/19a/155	100.0
B-NHL-NOS	100.0	100.0	100.0	100.0	85.7	-19a/155	100.0

Specificity

	miR-21	miR-19a	miR-20a	miR-92a	miR-155	Index	Percent
DLBCL	94.7	100.0	85.0	95.0	75.0	-21/19a/20a/155	85.0
MCL	100.0	100.0	90.0	95.0	100.0	-21/19a/155	95.0
BL	100.0	100.0	90.0	100.0	80.0	-21/19a/155	100.0
B-NHL-NOS	89.5	100.0	100.0	100.0	55.0	-19a/155	100.0

Youden index

	miR-21	miR-19a	miR-20a	miR-92a	miR-155	Index	Percent
DLBCL	90.4	83.3	76.7	76.8	54.2	-21/19a/20a/155	76.3
MCL	100.0	100.0	82.3	87.3	85.7	-21/19a/155	95.0
BL	100.0	80.0	70.0	80.0	55.0	-21/19a/155	100.0
B-NHL-NOS	89.5	100.0	100.0	100.0	40.7	-19a/155	100.0

Table S2. Sensitivity and specificity of oncomiRs in CSF and plasma of secondary CNS lymphoma patients compared to controls.

Percent of oncomiR sensitivity, specificity and Youdan index in CSF (**A**) and plasma (**B**) of indicated secondary CNS lymphoma compared to controls. The sensitivity and specificity is determined according to the minimal distance of the ROC curve to the top left corner of the plot square, evaluated by Receiver operating analysis. The Youden index is the sum of sensitivity and specificity subtracted by 100. **Left panel** represents individual oncomiR, **right panel**: oncomiR-index combining expression of individual oncomiRs into one classifier.

A CSF

CNS vs Systemic lymphoma - single miR / oncomiR-index

CSF – Sensitivity

CNS lymphoma	miR-21	miR-19a	miR-20a	miR-92a	miR-155	Index	Percent
DLBCL	83.3	75.0	75.0	83.3	72.0	-21/20a/155	91.3
MCL	93.3	80.0	80.0	85.7	87.5	-21/20a/92a/155	87.5
BL	100.0	60.0	80.0	60.0	100.0	-21/155	100.0
B-NHL-NOS	77.8	77.8	66.7	66.7	87.5	-21/155	88.9

CSF – Specificity

	miR-21	miR-19a	miR-20a	miR-92a	miR-155	Index	Percent
DLBCL	87.5	77.0	77.1	79.7	82.2	-21/20a/155	90.4
MCL	91.7	75.0	76.5	86.7	87.5	-21/20a/92a/155	93.3
BL	84.6	46.2	53.9	53.9	91.7	-21/155	100.0
B-NHL-NOS	66.7	66.7	66.7	66.7	100.0	-21/155	100.0

CSF – Youden index

	miR-21	miR-19a	miR-20a	miR-92a	miR-155	Index	Percent
DLBCL	70.8	52.0	52.1	63.0	54.2	-21/20a/155	81.7
MCL	85.0	55.0	56.5	72.4	75.0	-21/20a/92a/155	80.8
BL	84.6	6.2	33.9	13.9	91.7	-21/155	100.0
B-NHL-NOS	44.5	44.5	33.3	33.3	87.5	-21/155	88.9

B Plasma

Plasma – Sensitivity

	miR-21	miR-19a	miR-20a	miR-92a	miR-155	Index	Percent
DLBCL	78.3	79.2	79.2	77.3	62.5	-21/19a/20a/155	83.3
MCL	61.5	71.4	69.2	69.2	71.4	-21/19a/155	78.6
BL	100.0	80.0	80.0	80.0	75.0	-21/19a/155	100.0
B-NHL-NOS	66.7	66.7	71.4	71.4	57.1	-19a/155	100.0

Plasma – Specificity

	miR-21	miR-19a	miR-20a	miR-92a	miR-155	Index	Percent
DLBCL	74.6	79.6	78.3	77.6	65.0	-21/19a/20a/155	78.3
MCL	73.3	62.5	62.5	62.5	62.5	-21/19a/155	100.0
BL	100.0	76.9	61.5	92.3	69.2	-21/19a/155	100.0
B-NHL-NOS	66.7	66.7	66.7	66.7	66.7	-19a/155	66.7

Plasma – Youden index

	miR-21	miR-19a	miR-20a	miR-92a	miR-155	Index	Percent
DLBCL	52.8	58.8	57.5	54.9	27.5	-21/19a/20a/155	61.7
MCL	34.9	33.9	31.7	31.7	33.9	-21/19a/155	78.6
BL	100.0	56.9	41.5	72.3	44.2	-21/19a/155	100.0
B-NHL-NOS	33.3	33.3	38.1	38.1	23.8	-19a/155	66.7

Table S3. Sensitivity and specificity of oncomiRs in CSF and plasma of secondary CNS lymphoma compared to systemic lymphoma.

Percent of oncomiR sensitivity, specificity and Youden index in CSF (**A**) and plasma (**B**) of indicated secondary CNS lymphoma compared to systemic lymphoma. The sensitivity and specificity is determined according to the minimal distance of the ROC curve to the top left corner of the plot square, evaluated by Receiver operating analysis. The Youden index is the sum of sensitivity and specificity subtracted by 100. **Left panel** represents individual oncomiR, **right panel**: oncomiR-index combining expression of individual oncomiRs into one classifier.

	DLBCL			MCL			BL			B-NHL NOS			All diagnoses		
**Characteristics**	**SCNSL (n=25)**	**Systemic (n=72)**	**All patients (N=97)**	**SCNSL (n=15)**	**Systemic (n=19)**	**All patients (N=34)**	**SCNSL (n=5)**	**Systemic (n=13)**	**All patients (N=18)**	**SCNSL (n=9)**	**Systemic (n=4)**	**All patients (N=13)**	**SCNSL (n=54)**	**Systemic (n=108)**	**All patients (N=162)**
**Age, median (range), years**	59 (27-79)	61 (23-81)	61 (23-81)	65 (47-83)	66 (44-82)	65 (44-83)	62 (39-70)	47 (19-85)	54 (19-85)	63 (53-73)	48 (33-72)	61 (33-73)	62.5 (27-83)	62 (19-85)	62 (19-85)
<60	13 (52.0)	32 (44.4)	45 (46.4)	5 (33.3)	6 (31.6)	11 (32.4)	1 (20.0)	9 (69.2)	10 (55.6)	3 (37.5)	2 (50.0)	5 (41.7)	22 (41.5)	49 (45.4)	71 (43.8)
>=60	12 (48.0)	40 (55.6)	52 (53.6)	10 (66.7)	13 (68.4)	23 (67.6)	4 (80.0)	4 (30.8)	8 (44.4)	5 (62.5)	2 (50.0)	7 (58.3)	31 (58.5)	59 (54.6)	91 (56.2)
**Sex**															
Male	15 (60.0)	44 (61.1)	59 (60.8)	15 (100.0)	14 (73.7)	29 (85.3)	3 (60)	10 (76.9)	13 (72.2)	7 (77.8)	3 (75.0)	10 (76.9)	40 (74.1)	71 (65.7)	111 (68.5)
Female	10 (40.0)	28 (38.9)	38 (39.2)	0 (0.0)	5 (26.3)	5 (14.7)	2 (40)	3 (23.1)	5 (27.8)	2 (22.2)	1 (25.0)	3 (23.1)	14 (25.9)	37 (34.3)	51 (31.5)
**ECOG/PC**															
0-1	9 (36.0)	38 (52.8)	47 (48.4)	7 (46.7)	15 (78.9)	22 (64.7)	3 (60.0)	6 (46.1)	9 (50.0)	2 (25.0)	3 (75.0)	5 (41.7)	21 (39.6)	62 (57.4)	83 (51.6)
2-4	16 (64.0)	34 (47.2)	50 (51.6)	8 (53.3)	4 (21.1)	12 (35.3)	2 (40.0)	7 (53.8)	9 (50.0)	6 (75.0)	1 (25.0)	7 (58.3)	32 (60.4)	46 (42.6)	78 (48.4)
**Ann Arbor stage**															
I or II	4 (16.0)	17 (23.6)	21 (21.6)	0 (0.0)	3 (15.8)	3 (8.8)	0 (0.0)	0 (0.0)	0 (0.0)	1 (14.3)	2 (50.0)	3 (27.3)	5 (9.6)	22 (20.4)	27 (16.9)
III or IV	21 (84.0)	55 (76.4)	76(78.4)	15 (100.0)	16 (84.2)	31 (91.2)	5 (100.0)	13 (100.0)	18 (100.0)	6 (85.7)	2 (50.0)	8 (72.7)	47 (90.4)	86 (79.6)	133 (83.1)
**Extranodal sites, n**															
0-1	9 (36.0)	35 (48.6)	44 (45.4)	2 (13.3)	11 (57.9)	13 (38.2)	2 (40.0)	3 (23.1)	5 (27.8)	3 (37.5)	3 (75.0)	6 (50.0)	16 (30.2)	52 (48.1)	68 (42.2)
>1	16 (64.0)	37 (51.4)	53 (54.6)	13 (86.7)	8 (42.1)	21 (61.8)	3 (60.0)	10 (76.9)	13 (72.2)	5 (62.5)	1 (25.0)	6 (50.0)	37 (69.8)	56 (51.9)	93 (57.8)
**Involvement of kidney and/or adrenal glands**	8 (32.0)	11 (15.3)	19 (19.5)	1 (6.7)	0 (0)	1 (2.9)	0 (0.0)	4 (30.8)	4 (22.2)	0 (0.0)	0 (0.0)	0 (0.0)	9 (17.0)	15 (13.9)	24 (14.9)
**IPI**															
Low	3 (12.0)	14 (19.4)	17 (17.5)	0 (0.0)	3 (15.8)	3 (9.1)	0 (0.0)	0 (0.0)	0 (0.0)	0 (0.0)	2 (50.0)	2 (18.2)	3 (5.9)	19 (17.6)	22 (13.8)
Low-intermediate	2 (8.0)	13 (18.1)	15 (15.5)	2 (14.3)	3 (15.8)	5 (15.2)	1 (20.0)	2 (15.4)	3 (16.7)	2 (29.0)	1 (25.0)	3 (27.2)	7 (13.7)	19 (17.6)	26 (16.4)
High-Intermediate	4 (16.0)	17 (23.6)	21 (22.7)	3 (21.4)	9 (47.4)	12 (36.4)	2 (40.0)	4 (30.8)	6 (33.3)	2 (29.0)	0 (0.0)	2 (18.2)	11 (21.6)	30 (27.8)	41 (25.8)
High	16 (64.0)	28 (38.9)	44 (45.3)	9(64.3)	4 (21.0)	13 (39.4)	2 (40.0)	7 (53.8)	9 (50.0)	3 (42.0)	1 (25.0)	4 (36.4)	30 (58.8)	40 (37.0)	70 (44.0)
**CNS-IPI**															
Low (0-1)	2 (8.7)	13 (18.1)	15 (15.8)												
Intermediate (2-3)	4 (17.4)	27 (37.5)	31 (32.6)												
High (4-6)	17 (73.9)	32 (44.4)	49 (51.6)												
**MIPI**															
Low (<5.7)				1 (6.7)	4 (15.0)	5 (15.0)									
Intermediate (5.7-6.2)				3 (20.0)	7 (30.0)	10 (30.0)									
High (>6.2)				11 (73.3)	8 (55.0)	18 (55.0)									
**Non-GCB cell-of-origin**	10 (62.5)	33 (55.9)	43 (57.3)												
**Bone marrow involvement**	12 (48.0)	17 (23.3)	29 (29.6)	14 (87.5)	14 (70.0)	28 (77.8)	5 (100.0)	6 (46.2)	11 (61.1)	4 (50.0)	0 (0.0)	4 (36.4)	35 (64.8)	37 (33.9)	72 (44.2)
**Peripheral blood involvement**	6 (26.1)	6 (9.4)	12 (13.8)	12 (80.0)	12 (60.0)	24 (68.6)	2 (40.0)	0 (0.0)	2 (11.8)	3 (42.9)	0 (0.0)	3 (30.0)	23 (46.0)	18 (18.2)	41 (27.5)
**LDH (plasma) > Norm**	14 (66.7)	42 (60.0)	56 (60.8)	11 (84.6)	10 (52.6)	21 (65.6)	3 (60.0)	12 (92.3)	15 (83.3)	6 (75.0)	2 (50.0)	8 (66.7)	34 (72.3)	66 (61.1)	100 (64.7)
**Lactate (CSF) > Norm**	10 (55.6)	10 (20.4)	20 (29.9)	4 (44.4)	5 (33.3)	9 (37.5)	3 (50.0)	3 (25.0)	5 (31.3)	7 (87.5)	0 (0.0)	7 (70.0)	23 (59.0)	18 (23.1)	41 (35.0)
**Total protein level (CSF) > Norm**	8 (44.4)	12 (24.0)	20 (29.4)	4 (44.4)	3 (20.0)	7 (29.2)	3 (75.0)	2 (16.7)	5 (31.3)	5 (62.5)	0 (0.0)	5 (50.0)	20 (51.3)	17 (21.5)	37 (31.4)
**β2 microglobuline (Plasma) > Norm**	11 (68.8)	43 (61.8)	54 (63.5)	11 (100.0)	13 (72.2)	24 (82.8)	4 (80.0)	11 (91.7)	15 (88.2)	2 (28.6)	1 (33.3)	3 (30.0)	28 (71.8)	68 (66.2)	96 (69.1)
**Albumin (Plasma) < Norm**	7 (50.0)	14 (41.2)	21 (43.8)	3 (37.5)	9 (69.2)	12 (57.1)	1 (33.3)	7 (63.6)	8 (51.1)	3 (60.0)	0 (0.0)	3 (37.5)	14 (46.7)	36 (59.0)	50 (54.9)

Table S4. Summary of baseline clinical characteristics of CNS involving and systemic lymphoma patients. Data are presented as n (%). SCNSL, secondary CNS lymphoma. DLBCL, Diffuse large B-cell lymphoma. MCL, Mantle-cell lymphoma. BL, Burkitt´s lymphoma. B-NHL-NOS (not otherwise specified, high grade).

	DLBCL						MCL			B-NHL NOS		
Characteristics	Systemic to CNS relapse subsequent (n=7)	Systemic to CNS relapse all (n=13)	Systemic no CNS relapse (n=65)	SCNSL relapse subsequent (n=4)	SCNSL relapse all (n=8)	SCNSL no CNS relapse (n=10)	CNS relapse (n=4)	SCNSL no CNS relapse (n=11)	Systemic no CNS relapse (n=19)	CNS relapse (n=1)	SCNSL no CNS relapse (n=8)	Systemic no CNS relapse (n=4)
**Age, median(range), years**	46 (27-74)	55 (27-78)	61 (23-81)	71 (59-79)	66.5 (46-79)	61.5 (37-79)	77 (62-83)	63 (47-75)	66 (44-82)	58	60 (57-73)	48 (33-72)
<60	4 (57.1)	8 (61.5)	28 (43.1)	1 (25.0)	2 (25.0)	5 (50.0)	0 (0.0)	5 (45.5)	6 (31.6)	1 (100.0)	2 (28.5)	2 (50.0)
>=60	3 (42.9)	5 (38.5)	37 (56.9)	3 (75.0)	6 (75.0)	5 (50.0)	4 (100.0)	6 (54.5)	13 (68.4)	0 (0.0)	5 (71.5)	2 (50.0)
**Sex**												
Male	7 (100.0)	10 (76.9)	37 (56.7)	3 (75.0)	5 (62.5)	6 (60.0)	4 (100.0)	11 (100.0)	14 (73.7)	0 (0.0)	7 (87.5)	3 (75.0)
Female	0 (0.0)	3 (23.1)	28 (43.1)	1 (25.0)	3 (37.5)	4 (40.0)	0 (0.0)	0 (0.0)	5 (26.3)	1 (100.0)	1 (12.5)	1 (25.0)
**ECOG/PC**												
0-1	2 (28.6)	5 (38.5)	36 (55.4)	1 (25.0)	2 (25.0)	3 (30.0)	4 (100.0)	3 (27.3)	15 (78.9)	0 (0.0)	2 (28.5)	3 (75.0)
2-4	5 (71.4)	8 (61.5)	29 (43.6)	3 (75.0)	6 (75.0)	7 (70.0)	0 (0)	8 (72.7)	4 (21.1)	1 (100.0)	5 (71.5)	1 (25.0)
**Ann Arbor stage**												
I or II	0 (0.0)	3 (23.1)	17 (26.2)	0 (0.0)	0 (0.0)	1 (10.0)	0 (0.0)	0 (0.0)	3 (15.8)	0 (0.0)	1 (16.7)	2 (50.0)
III or IV	7 (100.0)	10 (76.9)	48 (73.8)	3 (200.0)	7 (100.0)	9 (90.0)	4 (100.0)	11 (100.0)	16 (84.2)	1 (100.0)	5 (83.3)	2 (50.0)
**Extranodal sites, n**												
0-1	2 (28.6)	7 (53.8)	33 (50.8)	2 (50.0)	2 (25.0)	2 (20.0)	1 (25.0)	1 (9.1)	11 (57.9)	0 (0.0)	3 (42.9)	3 (75.0)
>1	5 (71.4)	6 (46.2)	32 (49.2)	2 (50.0)	6 (75.0)	8 (80.0)	3 (75.0)	10 (90.9)	8 (42.1)	1 (100.0)	4 (57.1)	1 (25.0)
**Involvement of kidney and/or adrenal glands**	1 (16.7)	1 (7.7)	10 (15.4)	0 (0.0)	1 (14.3)	5 (50.0)	0 (0.0)	1 (9.1)	0 (0)	0 (0.0)	0 (0.0)	0 (0.0)
**IPI**												
Low	1 (14.3)	4 (30.8)	13 (20.0)	0 (0.0)	0 (0.0)	0 (0.0)	0 (0.0)	0 (0)	3 (15.8)	0 (0.0)	0 (0.0)	2 (50.0)
Low-intermediate	0 (0.0)	1 (7.7)	13 (20.0)	0 (0.0)	0 (0.0)	1 (10.0)	0 (0.0)	2 (18.2)	3 (15.8)	0 (0.0)	2 (33.3)	1 (25.0)
High-Intermediate	1 (14.3)	0 (0)	16 (24.6)	2 (66.7)	2 (28.6)	3 (30.0)	2 (66.7)	1 (9.1)	9 (47.4)	1 (100.0)	1 (16.7)	0 (0.0)
High	5 (71.4)	8 (61.5)	23 (35.4)	1 (33.3)	5 (71.4)	6 (60.0)	1 (33.3)	8 (72.7)	4 (21.0)	0 (0.0)	3 (50.0)	1 (25.0)
**CNS-IPI**												
Low(0-1)	0 (0.0)	2 (15.4)	13 (20.0)	0 (0.0)	0 (0.0)	0 (0.0)						
Intermediate (2-3)	1 (14.3)	3 (23.1)	26 (40.0)	1 (25.0)	1 (12.5)	2 (20.0)						
High (4-6)	6 (85.7)	8 (61.5)	26 (40.0)	3 (75.0)	7 (87.5)	8 (80.0)						
**MIPI**												
Low (<5.7)							0 (0)	1 (9.1)	4 (15.0)			
Intermediate (5.7-6.2)							0 (0)	3 (27.3)	7 (30.0)			
High (>6.2)							4 (100.0)	7 (63.6)	8 (55.0)			
**Non-GCB cell-of-origin**	3 (60.0)	8 (72.7)	30 (56.6)	0 (0.0)	1 (33.3)	3 (50.0)						
**Bone marrow involvement**	3 (42.9)	3 (23.1)	14 (21.5)	3 (75.0)	7 (87.5)	4 (40.0)	3 (75.0)	11 (100.0)	14 (70.0)	1 (100.0)	3 (37.5)	0 (0.0)
**Peripheral blood involvement**	0 (0.0)	0 (0.0)	12 (18.5)	3 (75.0)	5 (62.5)	3 (30.0)	2 (50.0)	10 (90.9)	12 (60.0)	1 (100.0)	2 (25.0)	0 (0.0)
**LDH (plasma) > Norm**	4 (80.0)	5 (50.0)	38 (58.5)	2 (50.0)	4 (57.1)	9 (90.0)	1 (50.0)	11 (100.))	10 (52.6)	1 (100.0)	5 (83.3)	2 (50.0)
**Lactate (CSF) > Norm**	2 (50.0)	5 (50.0)	8 (18.2)	2 (100.0)	6 (100.0)	3 (37.5)	2 (100.0)	2 (28.6)	5 (33.3)	1 (100.0)	6 (85.7)	0 (0.0)
**Total protein level (CSF) > Norm**	2 (50.0)	4 (40.0)	10 (22.2)	2 (100.0)	5 (83.3)	3 (37.5)	1 (50.0)	2 (28.6)	3 (20.0)	1 (100.0)	4 (57.1)	0 (0.0)
**β2 microglobuline (Plasma) > Norm**	3 (75.0)	2 (28.6)	40 (62.5)	1 (33.3)	3 (60.0)	9)90.0)	1 (100.0)	10 (100.0)	13 (72.2)	0 (0.0)	2 (50.0)	1 (33.3)
**Albumin (Plasma) < Norm**	1 (33.3)	4 (44.4)	13 (41.9)	1 (100.0)	2 (50.0)	3 (75.0)	2 (100.0)	3 (50.0)	9 (69.2)	0 (0.0)	3 (75.0)	0 (0.0)

Table S5. Baseline clinical characteristics of patients who developed CNS relapse compared with patients with no CNS relapse.
Data are presented as n (%). In DLBCL, the category of **Systemic to CNS relapse subsequent** denotes systemic DLBCL with samples collected at time of initial diagnosis (n=7), who subsequently relapsed/progressed to CNS. The category of **Systemic to CNS relapse all** contains in addition the “current” relapses (n=6), which were collected at the time of CNS relapse. The category **SCNSL relapse subsequent** denotes secondary CNS patients, with samples collected at time of diagnosis, who after achieving complete remission developed subsequently CNS relapse (n=4). The category **SCNSL relapse all** in addition contains SCNSL relapses collected at time of SCNSL relapse (n=4). Note: several DLBCL CNS relapses have samples from both, systemic diagnose and from CNS relapse, these patients are not duplicated in “relapse all” category. All relapses in MCL and B-NHL-NOS are relapses of systemic disease to CNS.

Risk Factor	HR	95% CI	*P*
**OncomiR high vs. low**	**9.4**	**1.8 - 49.9**	**0.0088**
**CNS-IPI**			
**high (vs. low)**	**4.9**	**0.88 - 26.7**	**0.0695**
**int. (vs. low)**	**4.8**	**0.08 - 283**	**0.4497**
**high (vs. int.)**	**4.8**	**1.7 - 21.5**	**0.0402**
**CNS-IPI / Oncomir**			
**both risk (vs. no risk)**	**31.9**	**4.7 - 219**	**0.0004**
**one risk (vs. no risk)**	**1.4**	**0.08 - 22.6**	**0.8278**
**both risks (vs. one risk)**	**12.1**	**2.1 - 69.5**	**0.0056**

Table S6. Predictive value of circulating oncomiRs in plasma for CNS relapse in DLBCL.
Risk for CNS relapse stratified by oncomiR levels in plasma (at time of diagnosis), CNS-IPI and CNS-IPI/oncomiR combined risk categories (univariate analysis). Abbreviations: CNS, central nervous system; IPI, International Prognostic Index; Int., intermediate; HR, hazard ratio. For details on risk stratification, see Suppl. methods.

A

CSF	*miR-21*	*miR-19a*	*miR-20a*	*miR-92a*	*miR-155*
**DLBCL**	1.83		1.31		1.78
**MCL**	1.36		0.83	1.30	1.84
**BL**	1.57				1.75
**B-NHL-NOS**	1.10				1.64

Plasma	*miR-21*	*miR-19a*	*miR-20a*	*miR-92a*	*miR-155*
**DLBCL**	0.07	0.03	6.93		0.43
**MCL**	2.42	0.15			1.25
**BL**	2.12	0.08			0.10
**B-NHL-NOS**		5.85			0.45

B

CSF	*miR-21*	*miR-19a*	*miR-20a*	*miR-92a*	*miR-155*
**DLBCL**	0.09		0.16		1.15
**MCL**	0.05		0.12	0.13	1.28
**BL**	0.08				1.59
**B-NHL-NOS**	0.07				1.54

Plasma	*miR-21*	*miR-19a*	*miR-20a*	*miR-92a*	*miR-155*
**DLBCL**	0.003	0.003	0.37		1.76
**MCL**	0.12	0.01			3.22
**BL**	0.18	0.02			1.04
**B-NHL-NOS**		0.40			4.00

Table S7. Coefficients for oncomiR-indices calculations.
The values stand for the coefficients calculated as described in the methods and supplementary methods sections. A) Equalized coefficients (let-7a/Ctrl/systemic) B) uneqvalized coefficients.

p 15-18 :

Figure S6. Receiver operating analysis of oncomiRs in CSF of CNS lymphoma compared to controls or systemic lymphoma.
Receiver operating characteristic (ROC) curves for the possibility of indicated oncomiRs in CSF to discriminate between CNS lymphoma and controls (left panels) and between CNS involving and systemic lymphoma (right panels) in: (**A**) DLBCL, (**B**) MCL, (**C**) BL and (**D**) B-NHL-NOS. The X-axes show the percent of specificity and the Y-axes denote percent of sensitivity (100-sesintivity).

p 19-22:

Figure S7. Receiver operating analysis of oncomiRs in plasma of CNS lymphoma compared to controls or systemic lymphoma.
Receiver operating characteristic (ROC) curves for the possibility of indicated oncomiRs in plasma to discriminate between CNS lymphoma and controls (left panels) and between CNS involving and systemic lymphoma (right panels) in: (**A**) DLBCL, (**B**) MCL, (**C**) BL and (**D**) B-NHL-NOS. The X-axes show the percent of specificity and the Y-axes denote percent of sensitivity (100-sesintivity).

Figure S6A. Receiver operating analysis of oncomiRs in CSF of CNS lymphoma compared to controls or systemic lymphoma.

DLBCL CSF

A) **DLBCL-SCNSL vs. CTRL** | **DLBCL-SCNSL vs. syst**

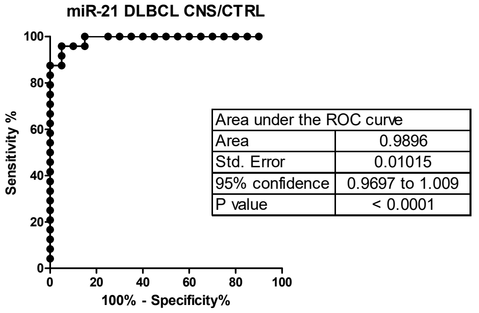

Area under the ROC curve	
Area	0.9896
Std. Error	0.01015
95% confidence	0.9697 to 1.009
P value	< 0.0001

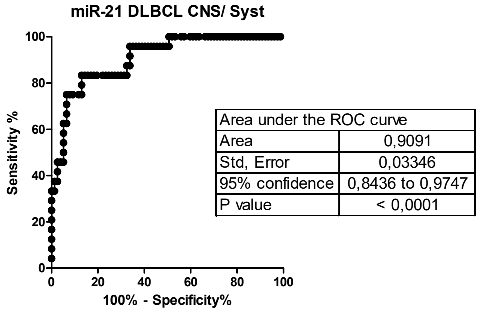

Area under the ROC curve	
Area	0,9091
Std, Error	0,03346
95% confidence	0,8436 to 0,9747
P value	< 0,0001

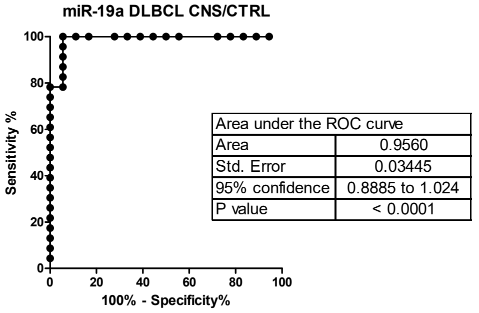

Area under the ROC curve	
Area	0.9560
Std. Error	0.03445
95% confidence	0.8885 to 1.024
P value	< 0.0001

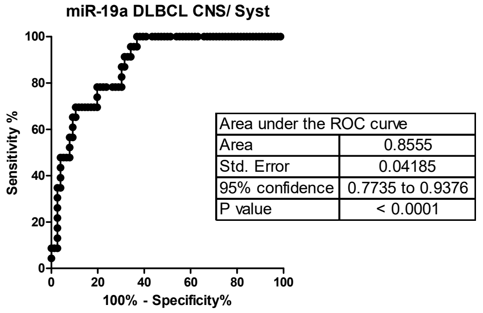

Area under the ROC curve	
Area	0.8555
Std. Error	0.04185
95% confidence	0.7735 to 0.9376
P value	< 0.0001

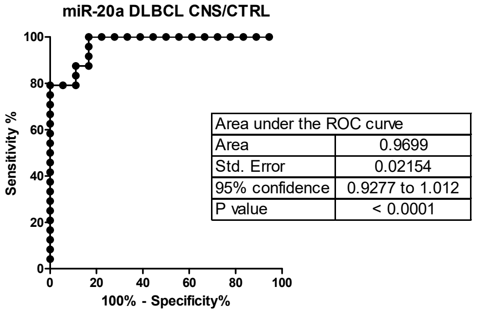

Area under the ROC curve	
Area	0.9699
Std. Error	0.02154
95% confidence	0.9277 to 1.012
P value	< 0.0001

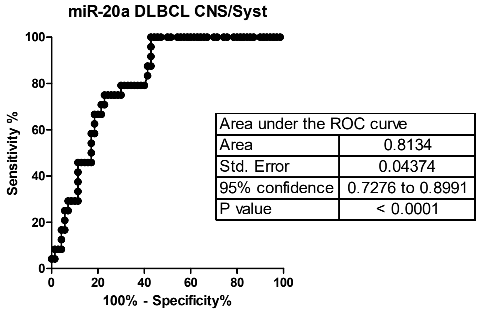

Area under the ROC curve	
Area	0.8134
Std. Error	0.04374
95% confidence	0.7276 to 0.8991
P value	< 0.0001

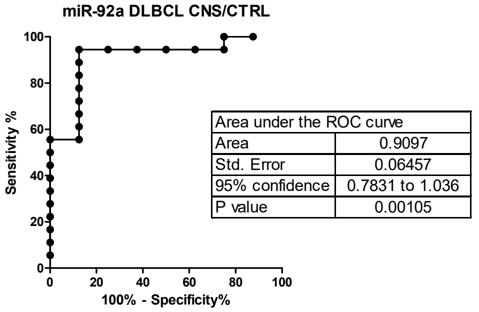

Area under the ROC curve	
Area	0.9097
Std. Error	0.06457
95% confidence	0.7831 to 1.036
P value	0.00105

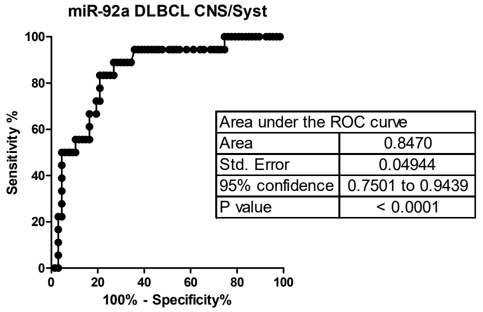

Area under the ROC curve	
Area	0.8470
Std. Error	0.04944
95% confidence	0.7501 to 0.9439
P value	< 0.0001

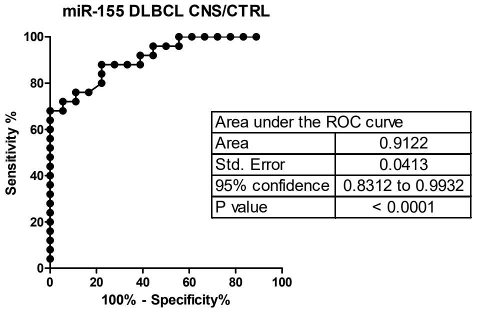

Area under the ROC curve	
Area	0.9122
Std. Error	0.0413
95% confidence	0.8312 to 0.9932
P value	< 0.0001

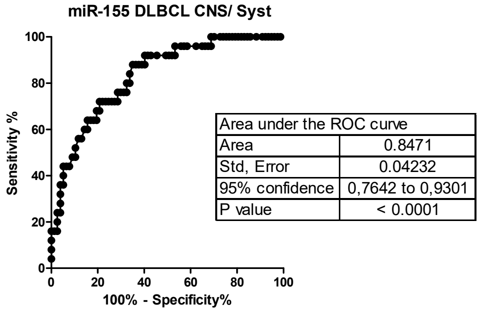

Area under the ROC curve	
Area	0.8471
Std, Error	0.04232
95% confidence	0,7642 to 0,9301
P value	< 0.0001

Figure S6B. Receiver operating analysis of oncomiRs in CSF of CNS lymphoma compared to controls or systemic lymphoma.

CSF MCL

B) **MCL-SCNSL vs. CTRL** **MCL-SCNSL vs. syst**

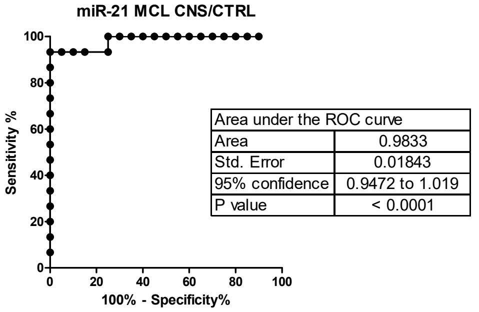

Area under the ROC curve	
Area	0.9833
Std. Error	0.01843
95% confidence	0.9472 to 1.019
P value	< 0.0001

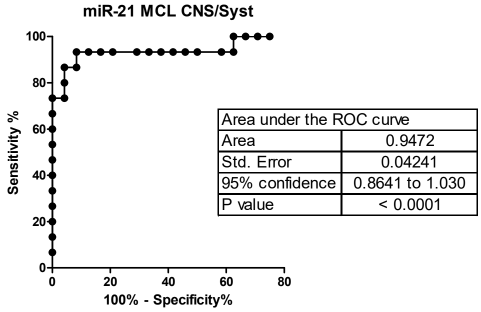

Area under the ROC curve	
Area	0.9472
Std. Error	0.04241
95% confidence	0.8641 to 1.030
P value	< 0.0001

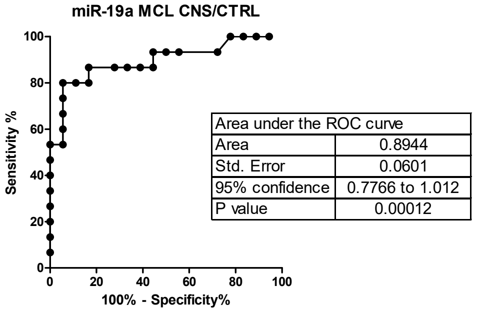

Area under the ROC curve	
Area	0.8944
Std. Error	0.0601
95% confidence	0.7766 to 1.012
P value	0.00012

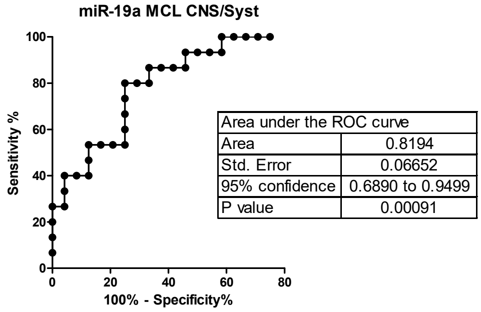

Area under the ROC curve	
Area	0.8194
Std. Error	0.06652
95% confidence	0.6890 to 0.9499
P value	0.00091

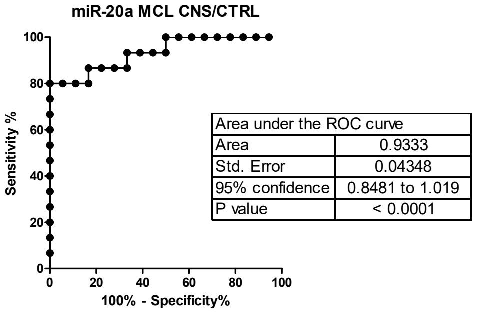

Area under the ROC curve	
Area	0.9333
Std. Error	0.04348
95% confidence	0.8481 to 1.019
P value	< 0.0001

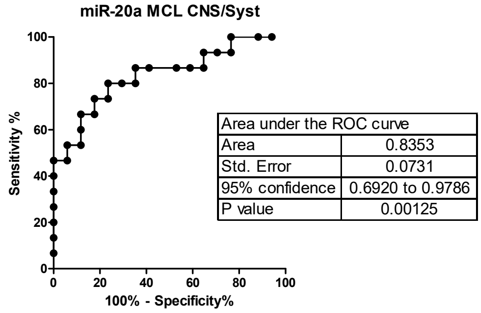

Area under the ROC curve	
Area	0.8353
Std. Error	0.0731
95% confidence	0.6920 to 0.9786
P value	0.00125

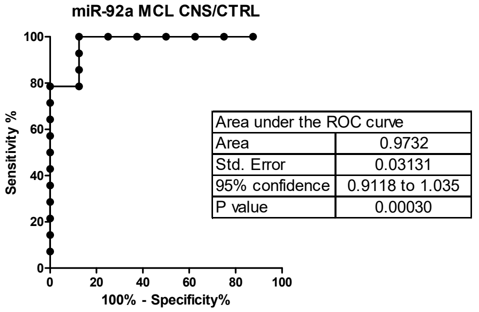

Area under the ROC curve	
Area	0.9732
Std. Error	0.03131
95% confidence	0.9118 to 1.035
P value	0.00030

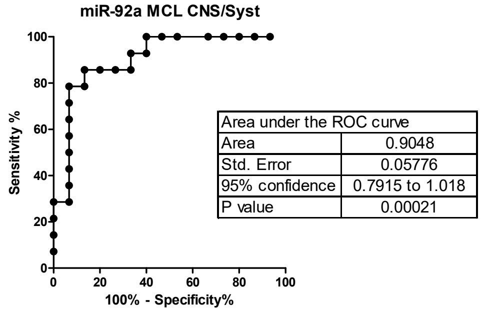

Area under the ROC curve	
Area	0.9048
Std. Error	0.05776
95% confidence	0.7915 to 1.018
P value	0.00021

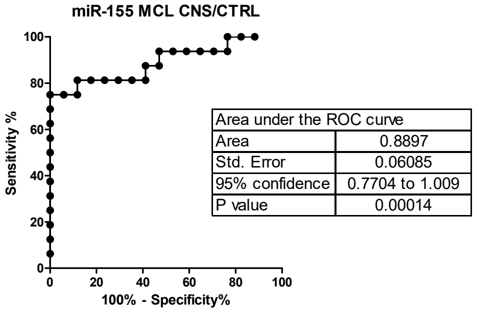

Area under the ROC curve	
Area	0.8897
Std. Error	0.06085
95% confidence	0.7704 to 1.009
P value	0.00014

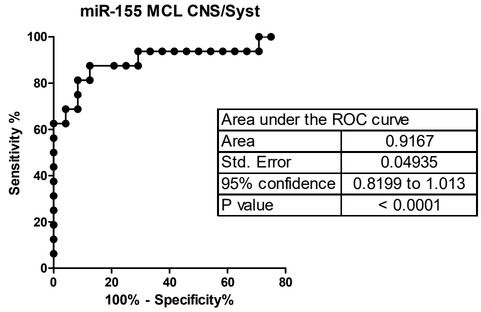

Area under the ROC curve	
Area	0.9167
Std. Error	0.04935
95% confidence	0.8199 to 1.013
P value	< 0.0001

Figure S6C. Receiver operating analysis of oncomiRs in CSF of CNS lymphoma compared to controls or systemic lymphoma.

CSF BL

C) **BL-SCNSL vs. CTRL** **BL-SCNSL vs. syst**

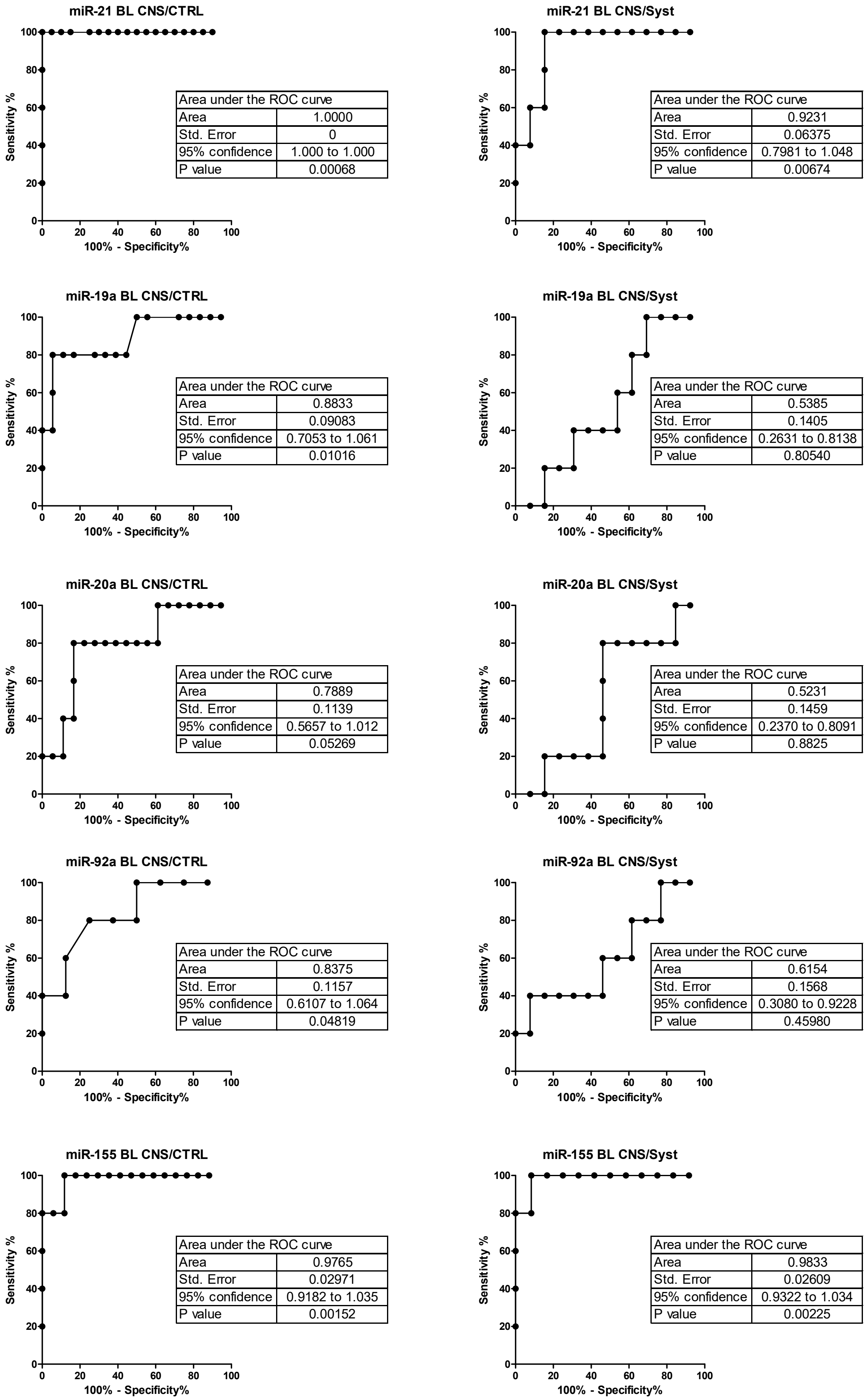

Figure S6D. Receiver operating analysis of oncomiRs in CSF of CNS lymphoma compared to controls or systemic lymphoma.

CSF B-NHL-NOS

D) **B-NHL NOS-SCNSL vs. CTRL** **B-NHL NOS-SCNSL vs. syst**

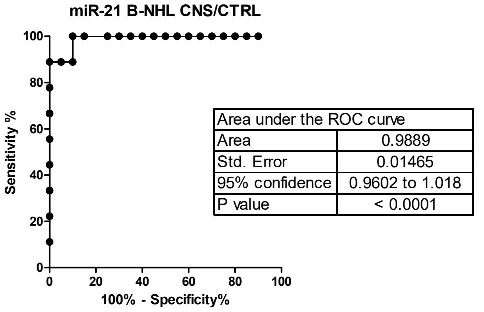

Area under the ROC curve	
Area	0.9889
Std. Error	0.01465
95% confidence	0.9602 to 1.018
P value	< 0.0001

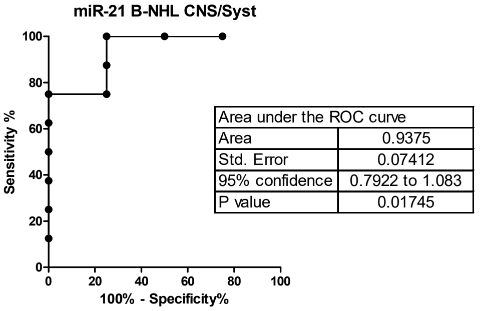

Area under the ROC curve	
Area	0.9375
Std. Error	0.07412
95% confidence	0.7922 to 1.083
P value	0.01745

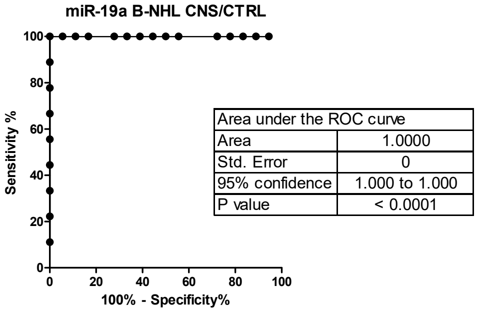

Area under the ROC curve	
Area	1.0000
Std. Error	0
95% confidence	1.000 to 1.000
P value	< 0.0001

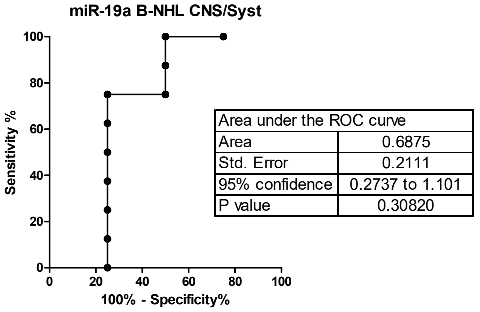

Area under the ROC curve	
Area	0.6875
Std. Error	0.2111
95% confidence	0.2737 to 1.101
P value	0.30820

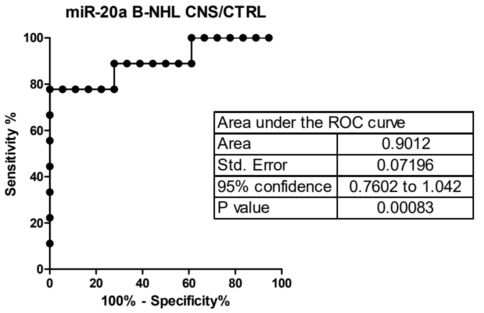

Area under the ROC curve	
Area	0.9012
Std. Error	0.07196
95% confidence	0.7602 to 1.042
P value	0.00083

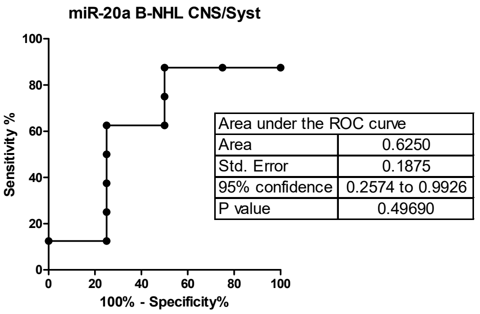

Area under the ROC curve	
Area	0.6250
Std. Error	0.1875
95% confidence	0.2574 to 0.9926
P value	0.49690

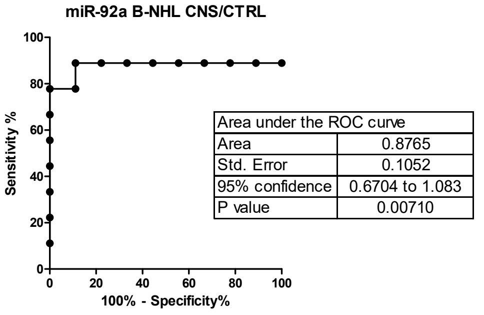

Area under the ROC curve	
Area	0.8765
Std. Error	0.1052
95% confidence	0.6704 to 1.083
P value	0.00710

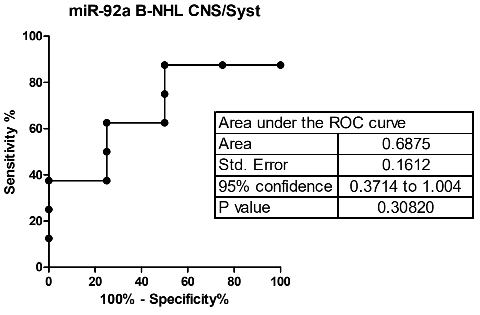

Area under the ROC curve	
Area	0.6875
Std. Error	0.1612
95% confidence	0.3714 to 1.004
P value	0.30820

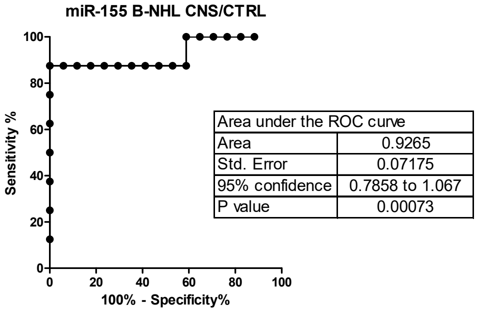

Area under the ROC curve	
Area	0.9265
Std. Error	0.07175
95% confidence	0.7858 to 1.067
P value	0.00073

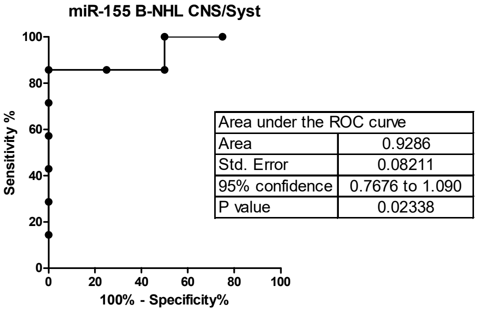

Area under the ROC curve	
Area	0.9286
Std. Error	0.08211
95% confidence	0.7676 to 1.090
P value	0.02338

Figure S7A. Receiver operating analysis of oncomiRs in plasma of CNS lymphoma compared to controls or systemic lymphoma.

Plasma DLBCL

A) DLBCL-SCNSL vs. CTRL | DLBCL-SCNSL vs. syst

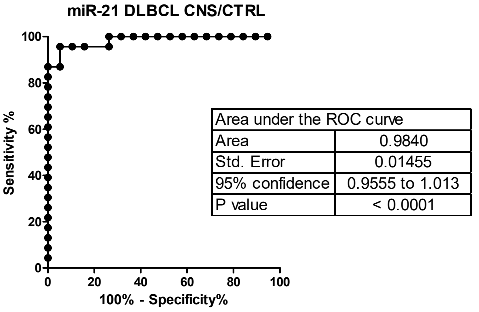

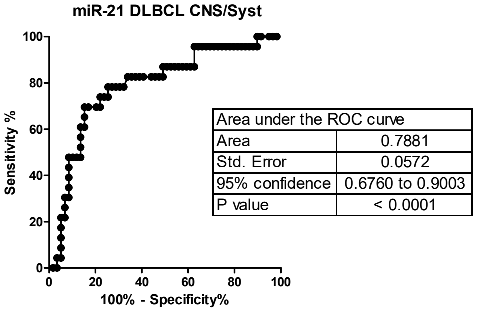

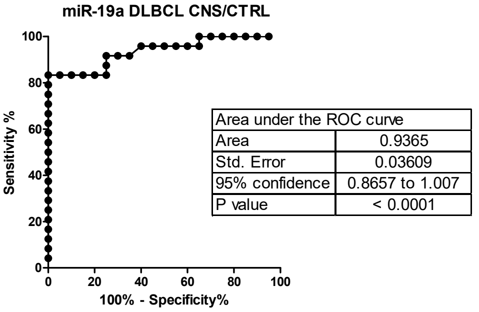

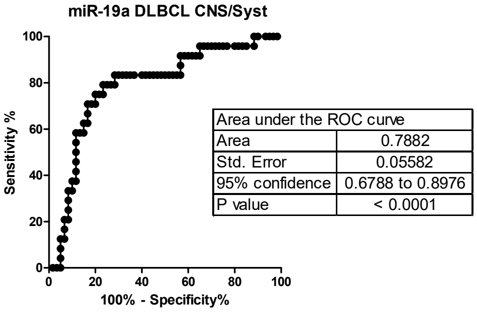

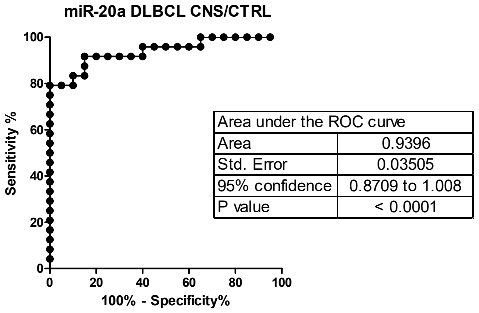

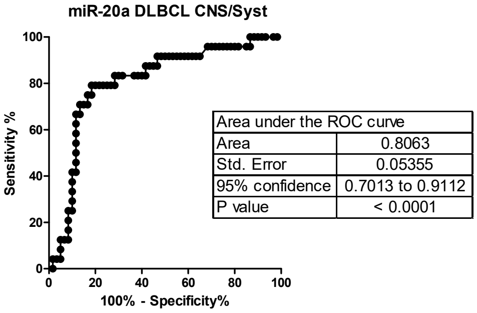

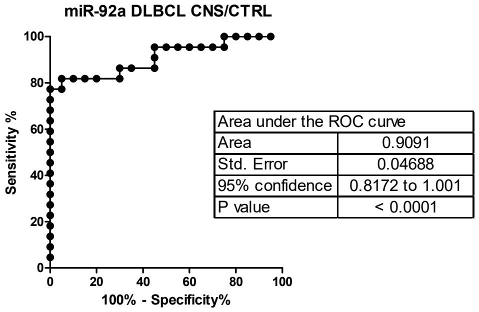

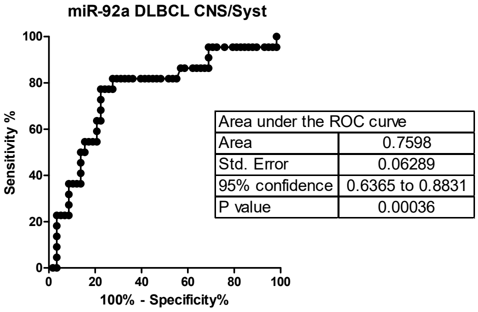

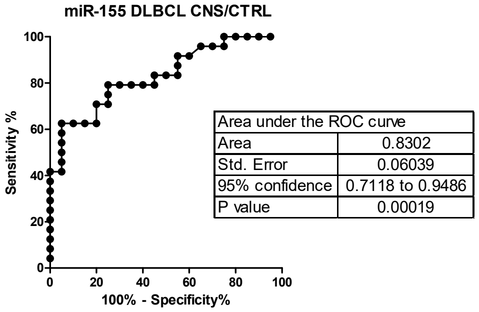

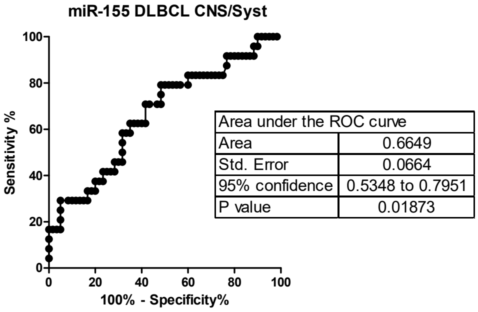

Figure S7B. Receiver operating analysis of oncomiRs in plasma of CNS lymphoma compared to controls or systemic lymphoma.

Plasma MCL

B) **MCL-SCNSL vs. CTRL** | **MCL-SCNSL vs. syst**

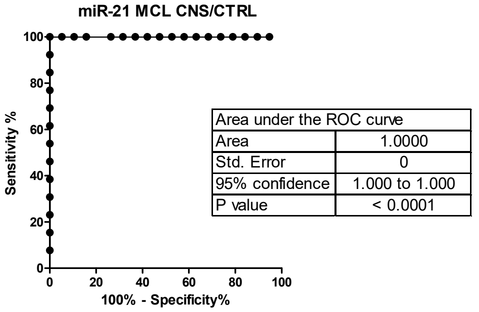

Area under the ROC curve	
Area	1.0000
Std. Error	0
95% confidence	1.000 to 1.000
P value	< 0.0001

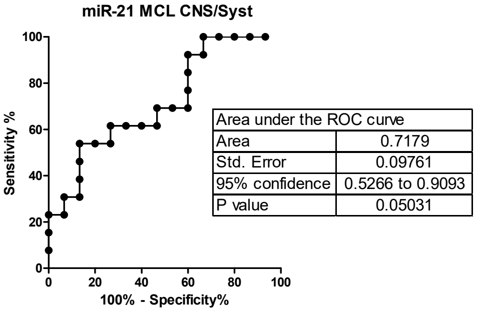

Area under the ROC curve	
Area	0.7179
Std. Error	0.09761
95% confidence	0.5266 to 0.9093
P value	0.05031

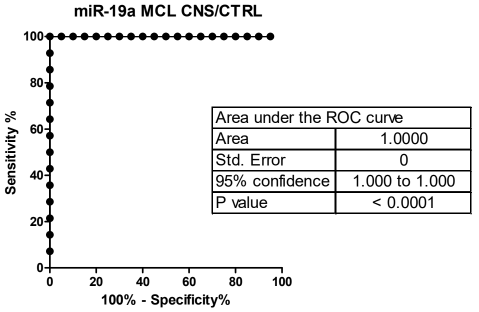

Area under the ROC curve	
Area	1.0000
Std. Error	0
95% confidence	1.000 to 1.000
P value	< 0.0001

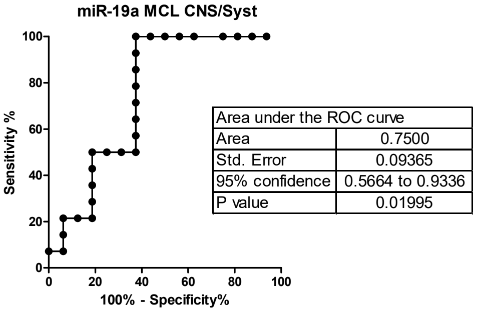

Area under the ROC curve	
Area	0.7500
Std. Error	0.09365
95% confidence	0.5664 to 0.9336
P value	0.01995

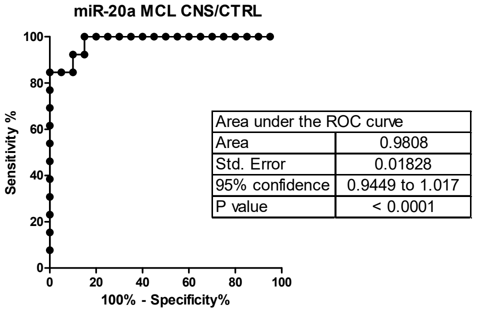

Area under the ROC curve	
Area	0.9808
Std. Error	0.01828
95% confidence	0.9449 to 1.017
P value	< 0.0001

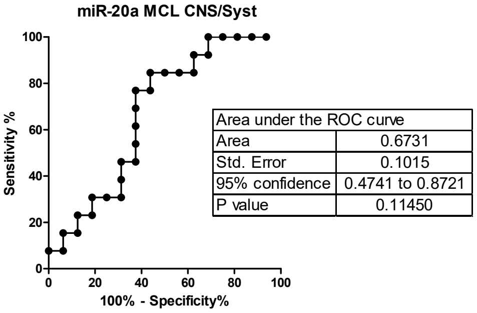

Area under the ROC curve	
Area	0.6731
Std. Error	0.1015
95% confidence	0.4741 to 0.8721
P value	0.11450

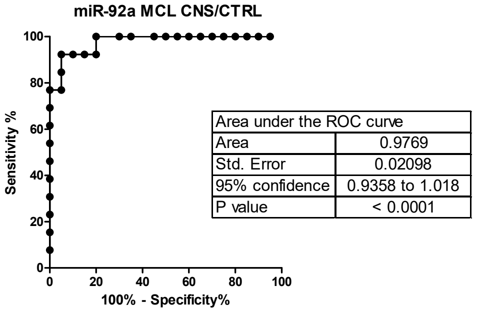

Area under the ROC curve	
Area	0.9769
Std. Error	0.02098
95% confidence	0.9358 to 1.018
P value	< 0.0001

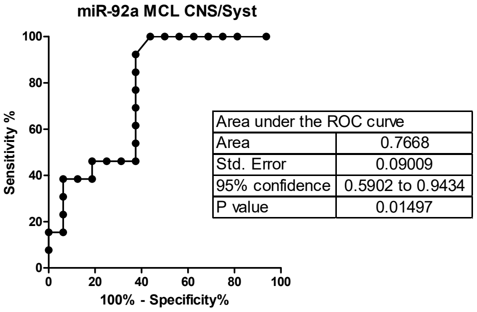

Area under the ROC curve	
Area	0.7668
Std. Error	0.09009
95% confidence	0.5902 to 0.9434
P value	0.01497

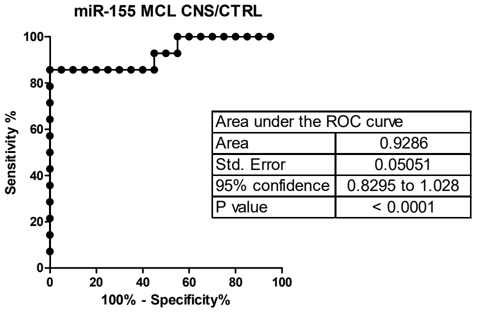

Area under the ROC curve	
Area	0.9286
Std. Error	0.05051
95% confidence	0.8295 to 1.028
P value	< 0.0001

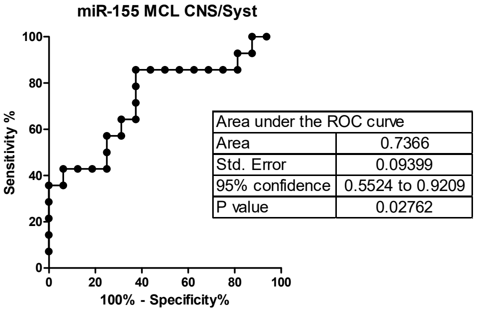

Area under the ROC curve	
Area	0.7366
Std. Error	0.09399
95% confidence	0.5524 to 0.9209
P value	0.02762

Figure S7C. Receiver operating analysis of oncomiRs in plasma of CNS lymphoma compared to controls or systemic lymphoma.

Plasma BL

C) BL-SCNSL vs. CTRL — **BL-SCNSL vs. syst**

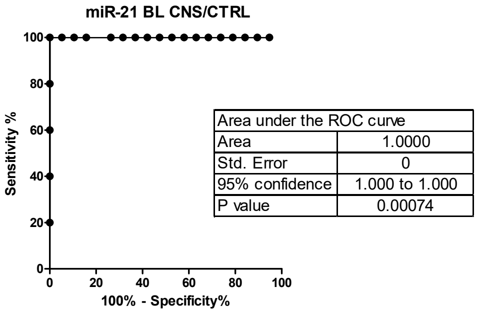

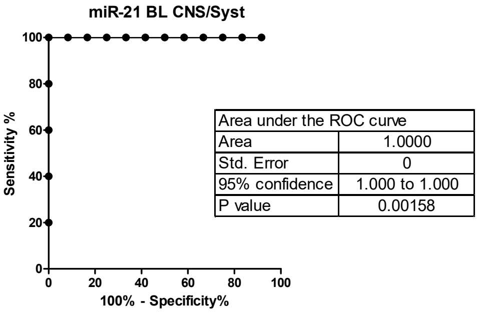

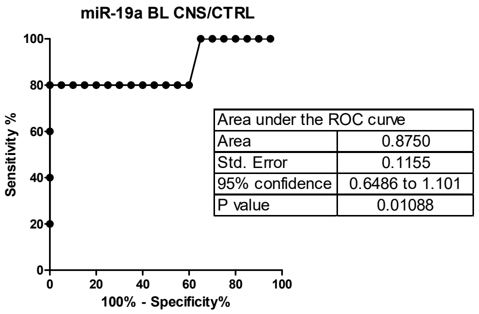

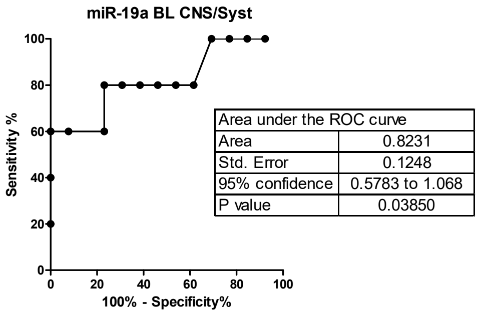

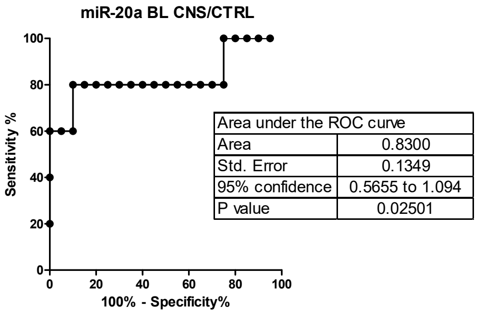

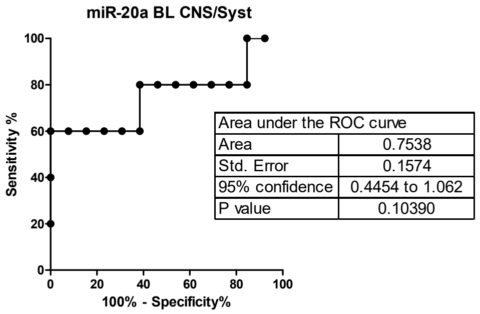

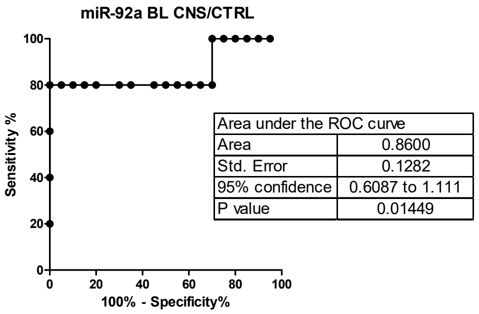

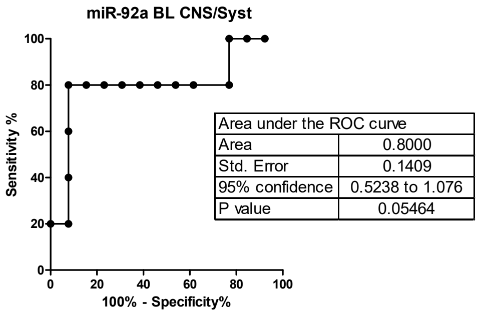

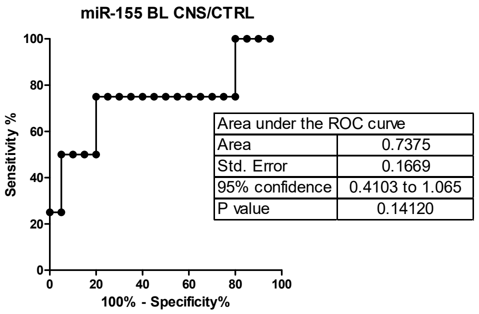

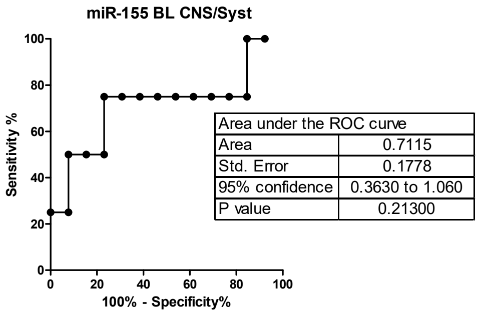

Figure S7D. Receiver operating analysis of oncomiRs in plasma of CNS lymphoma compared to controls or systemic lymphoma.

Plasma B-NHL-NOS

D) **B-NHL NOS-SCNSL vs. CTRL** **B-NHL NOS-SCNSL vs. syst**

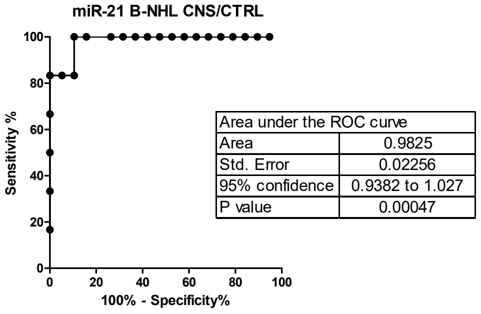

Area under the ROC curve	
Area	0.9825
Std. Error	0.02256
95% confidence	0.9382 to 1.027
P value	0.00047

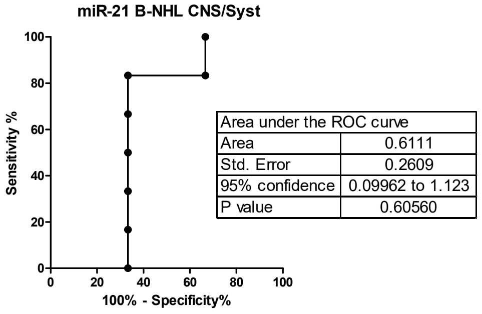

Area under the ROC curve	
Area	0.6111
Std. Error	0.2609
95% confidence	0.09962 to 1.123
P value	0.60560

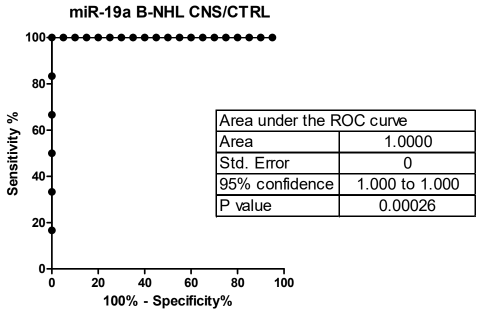

Area under the ROC curve	
Area	1.0000
Std. Error	0
95% confidence	1.000 to 1.000
P value	0.00026

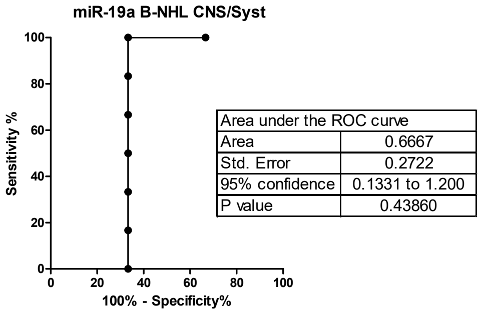

Area under the ROC curve	
Area	0.6667
Std. Error	0.2722
95% confidence	0.1331 to 1.200
P value	0.43860

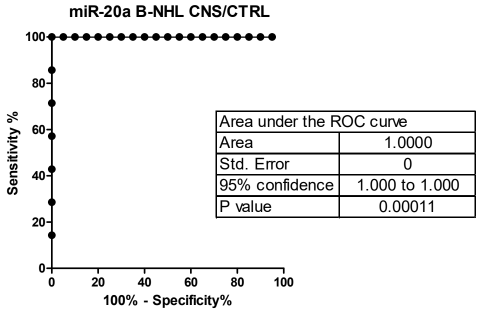

Area under the ROC curve	
Area	1.0000
Std. Error	0
95% confidence	1.000 to 1.000
P value	0.00011

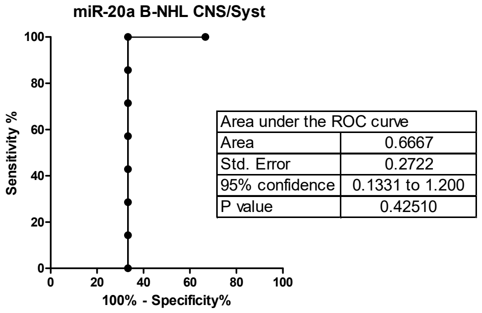

Area under the ROC curve	
Area	0.6667
Std. Error	0.2722
95% confidence	0.1331 to 1.200
P value	0.42510

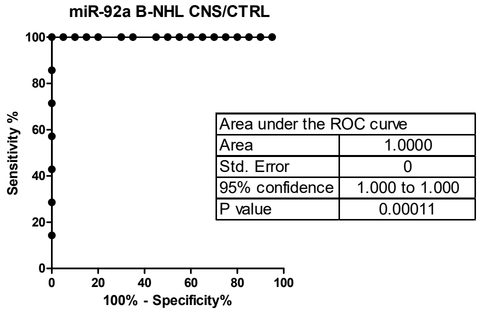

Area under the ROC curve	
Area	1.0000
Std. Error	0
95% confidence	1.000 to 1.000
P value	0.00011

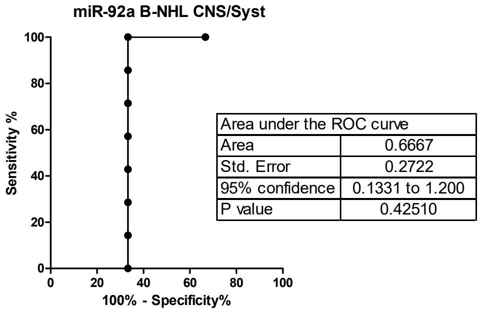

Area under the ROC curve	
Area	0.6667
Std. Error	0.2722
95% confidence	0.1331 to 1.200
P value	0.42510

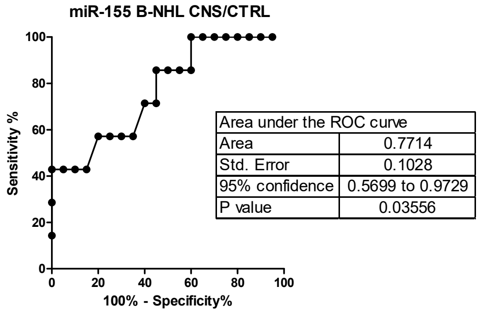

Area under the ROC curve	
Area	0.7714
Std. Error	0.1028
95% confidence	0.5699 to 0.9729
P value	0.03556

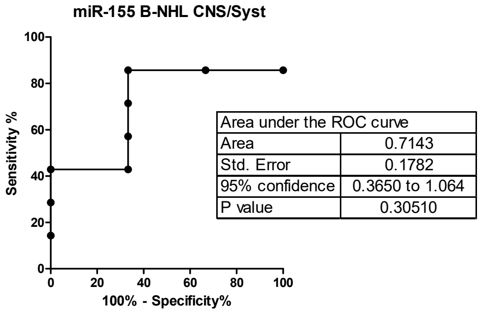

Area under the ROC curve	
Area	0.7143
Std. Error	0.1782
95% confidence	0.3650 to 1.064
P value	0.30510

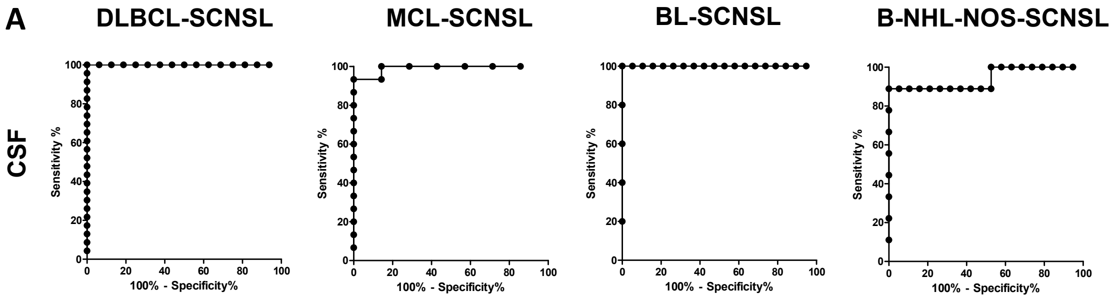

DLBCL-SCNSL (CSF)

Area under the ROC curve	
Area	1.00
Std. Error	0.0
95% confidence	1.00 to 1.00
P value	< 0.0001

MCL-SCNSL (CSF)

Area under the ROC curve	
Area	0.99
Std. Error	0.015
95% confidence	0.96 to 1.02
P value	0.00029

BL-SCNSL (CSF)

Area under the ROC curve	
Area	1.00
Std. Error	0.0
95% confidence	1.00 to 1.00
P value	0.00074

B-NHL-NOS-SCNSL (CSF)

Area under the ROC curve	
Area	0.94
Std. Error	0.058
95% confidence	0.83 to 1.06
P value	0.00021

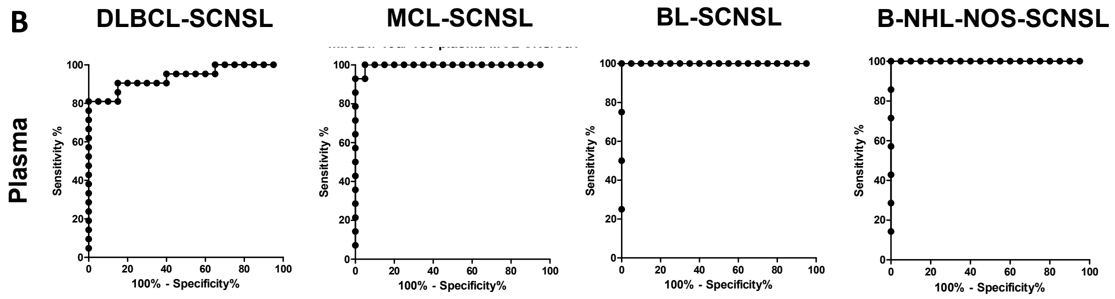

DLBCL-SCNSL (Plasma)

Area under the ROC curve	
Area	0.94
Std. Error	0.039
95% confidence	0.86 to 1.01
P value	< 0.0001

MCL-SCNSL (Plasma)

Area under the ROC curve	
Area	1.00
Std. Error	0.006
95% confidence	0.98 to 1.01
P value	< 0.0001

BL-SCNSL (Plasma)

Area under the ROC curve	
Area	1.00
Std. Error	0.0
95% confidence	1.00 to 1.00
P value	0.00196

B-NHL-NOS-SCNSL (Plasma)

Area under the ROC curve	
Area	1.00
Std. Error	0.0
95% confidence	1.00 to 1.00
P value	0.00011

Figure S8. Receiver operating analysis of oncomiR-indices in CSF (A) and plasma (B) of CNS lymphoma compared to control samples.
Receiver operating characteristic (ROC) curves for the possibility of indicated oncomiR-indices to discriminate between CNS lymphoma of indicated lymphoma subtypes and control samples. The X-axes show the percent of specificity and the Y-axes percent of sensitivity (100-sesintivity).
